# Investigating non-inferiority of internet-delivered versus face-to-face cognitive behavioural therapy for insomnia (CBT-I): a randomised controlled trial (iSleep well)

**DOI:** 10.1186/s13063-024-08214-6

**Published:** 2024-06-10

**Authors:** F. Benz, L. Grolig, S. Hannibal, C. Buntrock, P. Cuijpers, K. Domschke, D. D. Ebert, J. Ell, M. Janneck, C. Jenkner, A. F. Johann, A. Josef, M. Kaufmann, A. Koß, T. Mallwitz, H. Mergan, C. M. Morin, D. Riemann, H. Riper, S. R. Schmid, F. Smit, L. Spille, L. Steinmetz, E. J. W. Van Someren, K. Spiegelhalder, D. Lehr

**Affiliations:** 1 Department of Psychiatry and Psychotherapy, Medical Center - University of Freiburg, Faculty of Medicine, University of Freiburg, Freiburg, Germany; 2 Department of Health Psychology and Applied Biological Psychology, Institute of Sustainability Psychology, Leuphana University of Lüneburg, Lüneburg, Germany; 3 Institute of Social Medicine and Health Systems Research, Medical Faculty, Otto-von-Guericke-University, Magdeburg, Germany; 4 Department of Clinical, Neuro and Developmental Psychology, Amsterdam Public Health Research Institute, Vrije Universiteit Amsterdam, Amsterdam, Netherlands; 5 Babeș-Bolyai University, International Institute for Psychotherapy, Cluj-Napoca, Romania; 6 Psychology & Digital Mental Health Care, Department of Health Sciences, Technical University Munich, Munich, Germany; 7 Institute for Interactive Systems, Department of Electrical Engineering and Computer Science, Technische Hochschule Lübeck, Lübeck, Germany; 8 Clinical Trials Unit, Medical Center, Faculty of Medicine, University of Freiburg, Freiburg, Germany; 9 Institute of Medical Psychology and Medical Sociology, Faculty of Medicine, University of Freiburg, Freiburg, Germany; 10 École de Psychologie, Centre d’étude des troubles du sommeil, Centre de recherche CERVO/Brain Research Center, Université Laval, Québec, Canada; 11 Amsterdam UMC, Department of Psychiatry, Amsterdam Public Health Research Institute, Amsterdam, Netherlands; 12 Department of Epidemiology and Biostatistics, Amsterdam Public Health Research Institute, Amsterdam University Medical Centers, Location VUmc, Amsterdam, the Netherlands; 13 Centre of Health-Economic Evaluation, Trimbos Institute (Netherlands Institute of Mental Health and Addiction), Utrecht, the Netherlands; 14 Department of Sleep and Cognition, Netherlands Institute for Neuroscience, Royal Netherlands Academy of Arts and Sciences, Amsterdam, the Netherlands

**Keywords:** Insomnia, CBT-I, Cognitive behavioural therapy for insomnia, Internet-delivered, Digital, Face-to-face, Non-inferiority trial, Randomised controlled trial, Guided internet intervention

## Abstract

**Background:**

Insomnia is a highly prevalent disorder associated with numerous adverse health outcomes. Cognitive behavioural therapy for insomnia (CBT-I) is recommended as first-line treatment by clinical guidelines but is accessible to only a minority of patients suffering from insomnia. Internet-delivered CBT-I (iCBT-I) could contribute to the widespread dissemination of this first-line treatment. As there is insufficient evidence regarding non-inferiority, this study directly aims to compare therapist-guided internet-delivered versus face-to-face CBT-I in terms of insomnia severity post-treatment. Furthermore, a health-economic evaluation will be conducted, and potential benefits and disadvantages of therapist-guided iCBT-I will be examined.

**Methods:**

This study protocol describes a randomised controlled two-arm parallel-group non-inferiority trial comparing therapist-guided iCBT-I with face-to-face CBT-I in routine clinical care. A total of 422 patients with insomnia disorder will be randomised and treated at 16 study centres throughout Germany. Outcomes will be assessed at baseline, 10 weeks after randomisation (post), and 6 months after randomisation (follow-up). The primary outcome is insomnia severity measured using the Insomnia Severity Index. Secondary outcomes include depression-related symptoms, quality of life, fatigue, physical activity, daylight exposure, adverse events related to treatment, and a health-economic evaluation. Finally, potential moderator variables and several descriptive and exploratory outcomes will be assessed (e.g. benefits and disadvantages of internet-delivered treatment).

**Discussion:**

The widespread implementation of CBT-I is a significant healthcare challenge. The non-inferiority of therapist-guided iCBT-I versus face-to-face CBT-I will be investigated in an adequately powered sample in routine clinical care, with the same therapeutic content and same level of therapist qualifications provided with both interventions. If this trial demonstrates the non-inferiority of therapist-guided iCBT-I, healthcare providers may be more confident recommending this treatment to their patients, contributing to the wider dissemination of CBT-I.

**Trial registration:**

Trial registration number in the German Clinical Trials Register: DRKS00028153 (https://drks.de/search/de/trial/DRKS00028153). Registered on 16th May 2023.

**Supplementary Information:**

The online version contains supplementary material available at 10.1186/s13063-024-08214-6.

## Administrative information

Note: the numbers in curly brackets in this protocol refer to SPIRIT checklist item numbers. The order of the items has been modified to group similar items (see http://www.equator-network.org/reporting-guidelines/spirit-2013-statement-defining-standard-protocol-items-for-clinical-trials/).

**Table:** 

Title {1}	Investigating non-inferiority of internet-delivered versus face-to-face cognitive behavioural therapy for insomnia (CBT-I): a randomised controlled trial (iSleep well)
Trial registration {2a and 2b}.	Trial registration number: DRKS00028153, German Clinical Trials Register. Registered on 16 May 2023.
Protocol version {3}	28 February 2023, version 4.
Funding {4}	Funded by the German Research Foundation (Deutsche Forschungsgemeinschaft; DFG) – Projektnummer: 422022593.
Author details {5a}	F. Benz^1^*, L. Grolig^2^*, S. Hannibal^2^, C. Buntrock^3^, P. Cuijpers^4,5^, K. Domschke^1^, D.D. Ebert^6^, J. Ell^1^, M. Janneck^7^, C. Jenkner^8^, A.F. Johann^1,9^, A. Josef^8^, M. Kaufmann^8^, A. Koß^8^, T. Mallwitz^7^, H. Mergan^7^, C. M. Morin^10^, D. Riemann^1^, H. Riper^4,11^, S. R. Schmid^1^, F. Smit^4,12,13^, L. Spille^1^, L. Steinmetz^1^, E.J.W. Van Someren^14^, K. Spiegelhalder^1^*, and D. Lehr^2^* *These authors contributed equally (F. Benz and L. Grolig are shared first authors, K. Spiegelhalder and D. Lehr are shared last authors). ^1^Department of Psychiatry and Psychotherapy, Medical Center—University of Freiburg, Faculty of Medicine, University of Freiburg, Freiburg, Germany ^2^Department of Health Psychology and Applied Biological Psychology, Institute of Sustainability Psychology, Leuphana University of Lüneburg, Lüneburg, Germany ^3^Institute of Social Medicine and Health Systems Research, Medical Faculty, Otto-von-Guericke-University, Magdeburg, Germany Department of Clinical, Neuro and Developmental Psychology, Amsterdam Public Health Research Institute, Vrije Universiteit Amsterdam, Amsterdam, Netherlands ^5^Babeș-Bolyai University, International Institute for Psychotherapy, Cluj-Napoca, Romania ^6^Psychology & Digital Mental Health Care, Department of Health Sciences, Technical University Munich, Munich, Germany ^7^Institute for Interactive Systems, Department of Electrical Engineering and Computer Science, Technische Hochschule Lübeck, Lübeck, Germany ^8^Clinical Trials Unit, Medical Center, Faculty of Medicine, University of Freiburg, Freiburg, Germany ^9^Institute of Medical Psychology and Medical Sociology, Faculty of Medicine, University of Freiburg, Freiburg, Germany ^10^École de Psychologie, Centre d’étude des troubles du sommeil, Centre de recherche CERVO/Brain Research Center, Université Laval, Québec, Canada ^11^Amsterdam UMC, Department of Psychiatry, Amsterdam Public Health Research Institute, Netherlands ^12^Department of Epidemiology and Biostatistics, Amsterdam Public Health Research Institute, Amsterdam University Medical Centers, Location VUmc, Amsterdam, the Netherlands ^13^Centre of Health-Economic Evaluation, Trimbos Institute (Netherlands Institute of Mental Health and Addiction), Utrecht, the Netherlands ^14^Department of Sleep and Cognition, Netherlands Institute for Neuroscience, Royal Netherlands Academy of Arts and Sciences, Amsterdam, the Netherlands
Name and contact information for the trial sponsor {5b}	Department of Psychiatry and Psychotherapy, Medical Center—University of Freiburg, Faculty of Medicine, University of Freiburg, Freiburg, Germany, Hauptstraße 5, 79104 Freiburg
Role of sponsor {5c}	The main responsibility lies with the University Medical Center Freiburg, which assumes responsibilities in the capacity of sponsor. The sponsor is responsible for the design, management, and conduct of the trial, clinical monitoring of the trial, data analysis, and the dissemination of results. Furthermore, the sponsor ensures compliance with ethics bodies and legislation as well as with good clinical practices. The funder (DFG) has had and will have no involvement in study design, data collection or analysis, or the interpretation of results.

## Introduction

### Background and rationale {6a}

Insomnia disorder is defined as difficulties initiating and/or maintaining sleep and/or early morning awakening accompanied by daytime impairment [1]. To meet diagnostic criteria, the symptoms must occur at least three times per week and persist for a minimum of 3 months [1]. In Europe, approximately 10% of adults meet the criteria for insomnia disorder, with prevalence rates varying between 5.7 and 23.1% across different countries [2]. Insomnia is associated with reduced quality of life [3]. It also increases the risk of cardiovascular diseases [4, 5] and mental disorders, especially depression and anxiety disorders [6–8]. Furthermore, economic costs arise from both absenteeism and reduced productivity while at work (i.e., presenteeism) [9], along with increased healthcare utilisation [10, 11].

Clinical guidelines in Europe and the United States of America (USA) recommend cognitive behavioural therapy for insomnia (CBT-I) as first-line treatment for the disorder [12–15]. This multicomponent treatment consists of psychoeducation, sleep restriction therapy, stimulus control therapy, cognitive therapy, and relaxation techniques [16]. Unfortunately, treatment recommendations do not align with actual clinical practice. In reality, most patients with insomnia are prescribed hypnotic medication or sedating antidepressants [2], even though their long-term use is not recommended for insomnia, given side effects, risk of dependence or addiction, and questionable long-term efficacy [15].

Implementing CBT-I in routine clinical care poses a significant healthcare challenge. Internet-delivered CBT-I (iCBT-I) has the potential to make guideline-recommended therapy more accessible to people suffering from insomnia [17]. Besides improved accessibility, iCBT-I might have further advantages. For example, patients can complete the programme at their own pace, at any time and from anywhere with no travel expenses, and barriers associated with the stigma of mental disorders might be reduced [18, 19]. However, evidence on the non-inferiority of iCBT-I, relative to face-to-face treatment, remains insufficient.

Meta-analyses demonstrate moderate to large effect sizes for face-to-face CBT-I [20] and iCBT-I [21] compared to waitlist and non-active control groups with respect to sleep-related outcomes. Although results from these meta-analyses provide a useful estimate for comparative efficacy, conclusions are limited as the two CBT-I delivery formats were rarely compared directly within the same trial. To date, only limited evidence exists directly comparing face-to-face and internet-delivered CBT-I [22], including just few randomised controlled trials (RCTs) [23–26]. Findings from these studies are inconclusive with respect to the two treatment formats’ relative efficacy.

Two studies have sought to test the non-inferiority of iCBT-I, using either individual or group face-to-face CBT-I as comparison conditions. One of these trials investigated face-to-face group CBT-I by assuming a non-inferiority margin of 4 points on the Insomnia Severity Index (ISI; [27]) and confirmed the non-inferiority of therapist-guided iCBT-I in a sample of 48 patients [23]. However, it could be argued that the non-inferiority margin of 4 points on the ISI is too liberal, as a 4.6-point difference already indicates a clinically meaningful effect [28]. Furthermore, group-delivered therapy is not treatment-as-usual in Germany, as psychotherapy is predominantly provided in an individual treatment format. A stricter non-inferiority margin was applied in the second study by Kallestad et al. [25], using 2 points on the ISI. Here, the non-inferiority of iCBT-I without personal guidance by a therapist was not supported when compared to individual face-to-face CBT-I in a sample of 101 patients. This might be explained by the fully automated digital CBT-I condition, as other research indicates that iCBT-I should be provided with therapists’ support to achieve optimal results [29, 30] and greater acceptance by patients [29].

Two more trials compared the two therapy formats directly, though neither study specified a non-inferiority margin. In a trial by Lancee et al. [24], 30 patients were treated with face-to-face CBT-I by one experienced therapist, while another 30 patients received an iCBT-I programme with support from Masters-level students. On average, the face-to-face treatment led to an additional reduction in insomnia severity by 4.6 points, indicating a clinically meaningful advantage over iCBT-I [28]. In the second study, a trial among active duty military personnel that hypothesised the superiority of face-to-face CBT-I, a greater reduction on the ISI by 3 points was found relative to a newly developed, unguided iCBT-I approach [26]. The difference, however, was non-significant, which might be attributed to only 53% of the planned sample size being recruited, limiting the study’s statistical power.

Overall, evidence on the non-inferiority of iCBT-I is limited to date because sample sizes have been comparatively small (e.g. [23, 24]), and non-inferiority margins have either been not specified (e.g. [24, 26]) or questionably large (e.g. [23]). Moreover, therapists in each treatment arm had different levels of qualification [24], or therapist support was not considered at all [25]. Additionally, previous studies did not demonstrate that treatment content was equivalent [25] in the two treatment formats, and it is unclear whether treatments were conducted in settings representative of treatment in routine clinical care (e.g. [23, 24]). Therefore, consideration must be given to whether usual care is provided through group or individual CBT-I in the respective health systems to ensure the practical applicability of results.

Hence, there is a need for an RCT with rigorous methodology. Besides the limitations of previous clinical trials, numerous patients and practitioners continue to hold the belief that face-to-face treatments yield better outcomes than internet-delivered interventions, and there are concerns about the efficacy of the latter [31]. If a well-executed trial demonstrates the non-inferiority of iCBT-I, relative to face-to-face CBT-I, physicians and healthcare providers might refer their patients for iCBT-I with increased confidence and frequency, which would contribute to wider dissemination of this first-line insomnia treatment.

### Objectives {7}

This trial’s primary objective is to investigate the non-inferiority of therapist-guided iCBT-I versus face-to-face CBT-I using the primary endpoint, the Insomnia Severity Index (ISI; [27]) after treatment completion (10 weeks after randomisation). This will be investigated in an adequately powered sample in routine clinical care, ensuring the same therapeutic content and same therapist qualification level in both interventions. It is hypothesised that therapist-guided iCBT-I is non-inferior to individual face-to-face CBT-I. Secondary objectives of this trial are: (1) to perform a health-economic evaluation of the two interventions and (2) to systematically explore potential benefits and disadvantages associated with iCBT-I.

### Trial design {8}

This study—a randomised controlled non-inferiority trial conducted with two parallel groups (allocation ratio 1:1)—will compare therapist-guided iCBT-I and traditional face-to-face CBT-I.

### Methods: patients, interventions and outcomes

#### Study setting {9}

Patient recruitment started in June 2023 and is taking place in community and academic hospitals and outpatient clinics in Germany specialised in sleep medicine, psychiatry, psychosomatic medicine, or clinical psychology. Ethics approval for the study was obtained from the Ethics Committee of the University of Freiburg Medical Centre. The trial has been registered at the German Clinical Trials Register (DRKS00028153).

#### Eligibility criteria {10}

Inclusion criteria are as follows: (a) age ≥ 18 years; (b) DSM-5 diagnosis of insomnia disorder; (c) access to a computer and the internet; (d) home address not further than a 1-h commute to the nearest study centre; (e) fluent use of German; (f) written informed consent for study participation.

Exclusion criteria are as follows: (a) history of an additional sleep disorder (e.g. moderate or severe sleep apnoea syndrome, restless legs syndrome, narcolepsy); (b) severe or unstable psychiatric disorders (organic, including symptomatic, mental disorders (ICD-10: F00-F09), substance use disorders (ICD-10: F10-F19), schizophrenia, schizotypal and delusional disorders (ICD-10: F20-F29), severe major depressive disorder (ICD-10: F32.2, F32.3, F33.2, F33.3), or other psychiatric disorders clinically assessed as severe) or medical conditions requiring immediate treatment that can impact outcome variables or may be negatively affected by sleep restriction therapy (e.g. epilepsy, bipolar disorder, NREM parasomnia); (c) acute suicidality; (d) pregnancy; (e) ongoing psychotherapy or being on a waiting list for psychotherapeutic treatment; (f) participation in another clinical trial up to 30 days before or during participation in this study.

#### Who will take informed consent? {26a}

During the recruitment period, 16 study centres will recruit participants from patients seeking treatment for insomnia at these centres. Here, potentially eligible patients will receive verbal and written information about the study through a study therapist.

The screening procedure (~ 45 min) includes a face-to-face semi-standardised interview consisting of a detailed sleep history, as well as screening questions regarding psychiatric and medical conditions. In addition, all current sleep and psychopharmacological medications will be recorded. Written informed consent is mandatory for study participation. Consent can be withdrawn at any time without providing a reason.

#### Additional consent provisions for collection and use of patient data and biological specimens {26b}

Patients will be asked to provide their optional additional consent for merging their data with data from a survey with the therapists. In this survey, therapist variables (e.g. experience with iCBT-I, potential benefits and disadvantages of internet-delivered therapy for their therapeutic work) will be collected and examined as potential predictors of treatment effects. This informed consent is entirely voluntary. Patients opting to participate will receive identical treatment as those who choose not to give their consent. Refusal to grant consent will not result in any disadvantages for the patients. No biological specimens will be collected.

### Interventions

#### Explanation for the choice of comparators {6b}

In this non-inferiority trial, the control group will receive the first-line evidence-based treatment for insomnia in Germany, which is face-to-face CBT-I [15]. Therefore, the requirements of the Declaration of Helsinki are fulfilled, which states that “the benefits, risks, burdens, and effectiveness of a new intervention must be tested against those of the best proven intervention” [32].

### Intervention description {11a}

Both therapist-guided iCBT-I and face-to-face CBT-I are based on a published and widely used German-language CBT-I manual [33]. Thus, the two formats are identical in content and encompass the following CBT-I core components: (a) psychoeducation, (b) sleep restriction therapy, (c) stimulus control therapy, (d) cognitive therapy, and (e) relaxation techniques. Both formats consist of six sessions lasting 50 min each. The CBT-I components are delivered in the same order. In both groups, treatment is considered to be in accordance with the protocol if (1) at least 4 of the 6 scheduled sessions took place within the specified 10 weeks after randomisation, (2) no additional psychotherapeutic treatment was sought, and (3) there has been no increase in psychiatric medication. All therapists will be certified psychological or psychiatric psychotherapists for behavioural therapy with expertise in sleep medicine, or psychotherapy trainees supervised by certified psychological or psychiatric psychotherapists for behavioural therapy with expertise in sleep medicine, reflecting the current situation of insomnia treatment in Germany. All therapists will provide both treatments. To facilitate a standardised procedure and the equivalence of content, treatment manuals for both CBT-I conditions will be used by therapists, comprising templates and boilerplates (therapist-guided iCBT-I) and example formulations for psychoeducational information plus exercise sheets (face-to-face CBT-I). Table 1 summarises the content of the six sessions, which will be the same for both treatment conditions.

**Table 1:** Session content of the face-to-face and therapist-guided iCBT-I

Session	Main objectives	Content / exercises
1. My good start	• Receiving an overview of the therapy (face-to-face or internet-delivered); defining therapy goals; learning about sleep and insomnia	• Psychoeducation on sleep & insomnia • Defining **p**recipitating, **p**redisposing, and **p**erpetuating factors (3-P model) • Learning sleep hygiene rules • Becoming familiar with the sleep diary (app) • Implementing small and larger relaxation activities in everyday life
2. Renewing the sleep–wake rhythm	• Being introduced to the behavioural components of CBT-I	• Reviewing the relaxation and sleep hygiene activities • Understanding sleep restriction and stimulus control and how to combine them • Determining the bedtime window • Learning tips and tricks for implementing sleep restriction and stimulus control
3. Strengthening the sleep schedule	• Dealing with adverse effects and challenges of sleep restriction and stimulus control; maintaining motivation	• Reviewing implementation of sleep restriction and stimulus control • Adjusting the bedtime window • Dealing with adverse effects and handling challenges • Motivating to keep going • Introducing the first relaxation technique: Progressive Muscle Relaxation (PMR)
4. Dealing with worries and ruminations	• Learning how to deal with sleep-disturbing thoughts and ruminations	• Reviewing implementation of sleep restriction and stimulus control & adjusting the bedtime window • Identifying and challenging dysfunctional beliefs about sleep • Introducing two cognitive techniques: worry chair; thought diary • Introducing a second relaxation technique: body scan
5. Letting go of worries and ruminations	• Trying out a metacognitive technique and another relaxation technique	• Reviewing implementation of sleep restriction and stimulus control & adjusting the bedtime window • Reviewing cognitive techniques’ implementation • Introducing a third cognitive technique: allowing thoughts to come and go (imagination technique) • Introducing a third relaxation technique: mental image of calmness
6. Empowered into the future	• Consolidating and looking back at what has been learned; making a plan for the future	• Reviewing implementation of sleep restriction and stimulus control & adjusting the bedtime window • Reviewing experiences and changes over the past weeks • Relapse prevention: making a plan regarding which methods will be pursued further • Answering open questions

The therapist-guided iCBT-I will be delivered via an access-controlled online platform [34] designed as an interactive website that includes video clips, written information, graphical illustrations, written and audio exercises, examples of other (fictional) patients, and an app-based sleep diary. It is a revised version of an intervention initially developed for workers suffering from insomnia, which we adapted to the routine care setting. The original intervention has been positively evaluated in three randomised controlled trials [35–37]. Therapists have access to their patients’ data and provide written feedback after each completed session (e.g. validating exercises patients completed within the sessions, motivating patients to implement exercises, like sleep restriction therapy, in their daily lives and/or providing additional information). Therapists are advised to provide feedback within two working days and to spend no more than 60 min for writing their feedback for one session.

In the face-to-face CBT-I condition, therapists and patients together work out the CBT-I components. For example, therapists provide information (e.g. psychoeducation about sleep and insomnia) and introduce exercises (e.g. sleep restriction therapy) that patients will be asked to implement in their daily lives. Psychoeducational elements, therapy exercises, and media (e.g. audio relaxation exercises) are the same as those used with the iCBT-I. Patients receive a paper and pencil sleep diary, which is commonly used in routine care CBT-I.

### Criteria for discontinuing or modifying allocated interventions {11b}

One or more of the following circumstances may lead to discontinuation of intervention participation for an individual patient: (a) withdrawal of the patient’s consent, (b) loss of contact with the patient, (c) death of the patient, or (d) other unforeseeable circumstances that would endanger the patient’s health if they continued to participate in the intervention.

### Strategies to improve adherence to interventions {11c}

Adherence—encompassing each patient’s implementation of CBT-I elements and general session attendance—will be documented by therapists in both intervention arms. In cases of non-attendance at a session, therapists will either send written reminders (iCBT-I), call patients by telephone, or send an email (face-to-face CBT-I).

### Relevant concomitant care permitted or prohibited during the trial {11d}

Patients will be asked not to begin any further psychotherapy or new psychiatric/sleep medication, and not increase their psychiatric/sleep medication during study participation. If patients begin another psychotherapy or begin/increase psychiatric/sleep medication during study participation, they may continue with the intervention they were allocated to, but will not be included in the per-protocol analysis (see section “Statistical methods for primary and secondary outcomes {20a}”).

### Provisions for post-trial care {30}

As CBT-I is usually highly effective, no special services are planned after study participation. Patients are encouraged to contact their study centre in case of adverse events or if post-trial care is needed (e.g. non-remitters, non-responders, dropouts).

### Outcomes {12}

**Fig. 1:**
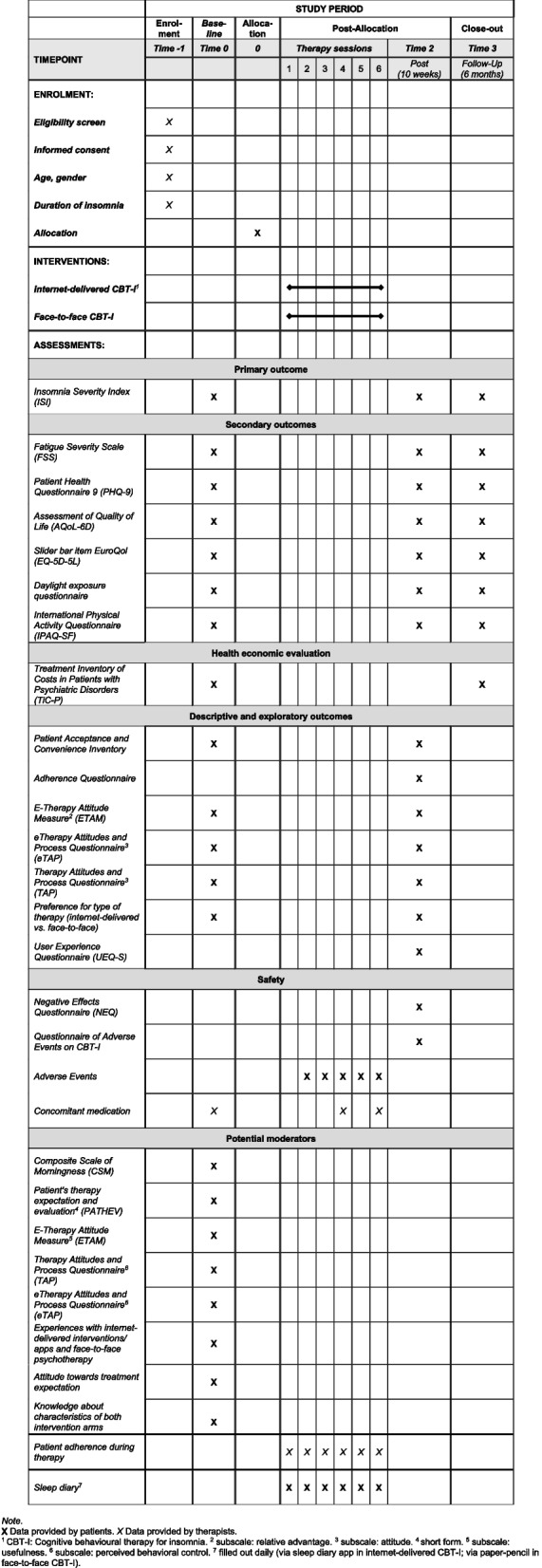
Study schedule of enrolment, interventions, and assessments

An overview of primary, secondary, economic, and exploratory outcomes is available in Fig. 1.

#### Primary outcome

The primary study outcome will be insomnia severity at post-intervention, assessed using the Insomnia Severity Index (ISI; [27]), a questionnaire that is commonly used in insomnia research [20, 29, 38] and clinical care [15]. Insomnia severity will be assessed at all measurement points (baseline, post-intervention 10 weeks after randomisation, and follow-up 6 months after randomisation). The ISI questionnaire consists of seven items, each answered on a 5-point Likert scale with response options ranging from 0 to 4 and a total score ranging from 0 to 28. In several studies, the ISI has demonstrated good internal consistency with *α* ranging from 0.70 to 0.90 [27, 28, 39]. For all measurement points, total scores will be calculated.

#### Secondary outcomes

Secondary outcomes will be assessed at all measurement points (baseline, post-intervention 10 weeks after randomisation, and follow-up 6 months after randomisation).

Fatigue severity will be measured using the Fatigue Severity Scale (FSS; [40]), comprised of nine items, each rated on a 7-point Likert scale ranging from 1 to 7. The total score ranges from 9 to 63. Internal consistency is good, with *α* = 0.88 [40].

Depression-related symptom severity will be measured using the 9-item version of the Patient Health Questionnaire (PHQ-9; [41]), with response options ranging from 0 to 3, a total score from 0 to 27, and a good *α* of 0.89.

Health-related quality of life will be assessed using the Assessment of Quality of Life (AQoL-6D; [42]), a questionnaire comprised of 20 items that measure six domains (independent living, pain, senses, mental health, coping, relationships). A total score can be calculated across all domains, ranging from 20 to 99. The domains’ reliability ranges from *α* = 0.50 (senses) to *α* = 0.86 (independent living; [42]). Patients’ health (range 0 to 100) will be measured using the slider bar item of the European Quality of Life 5 Dimensions 5 Level questionnaire (EQ-5D-5L [43]).

Daylight exposure will be assessed with a self-developed 5-item questionnaire adapted from the item on daylight exposure of the Munich Chronotype Questionnaire [44]. These five items assess how much time patients have been spending in daylight, on average, at five different time periods during the day (in the early morning (before 8.00 a.m.), later morning (8.00–11.59 a.m.), noon (12.00 p.m. to 2.59 p.m.), afternoon (3.00 p.m. to 5.59 p.m.), evening (6.00 p.m. and after)) over the past 7 days. Total time spent in daylight will also be calculated (see Appendix 1 for further details).

Patients’ physical activity level (i.e., time spent in vigorous- and moderate-intensity activity, walking, and sedentary activity) will be measured using the International Physical Activity Questionnaire – Short Form (IPAQ-SF; [45]). The IPAQ-SF is comprised of seven items that ask individuals to indicate both the number of days and average time over the past week that they have spent at each activity level. A total physical activity level will be estimated by weighting the reported minutes per week within each activity category by a metabolic equivalent (MET) energy expenditure estimate assigned to each activity category (e.g. cycling at vigorous intensity = 8, walking at moderate intensity = 3.3, sitting = 1; see [45]). The weighted MET-minutes per week will be calculated as duration × frequency per week × MET intensity. Test–retest reliability has been found to be very good, with Spearman’s *ρ* = 0.80 [45].

#### Health-economic evaluation

Quality-adjusted life years (QALYs) will be based on the AQoL-6D, which generates patient preference-based utilities on a scale of 0 (death) to 1 (perfect health), using the time-trade-off method [46]. QALY gains will be estimated by calculating the area under the curve (AUC) of linearly interpolated AQoL-6D utilities between measurement points to cover the follow-up period. Healthcare use, patient and family expenditures, and productivity losses will be self-assessed with the Trimbos and Institute for Medical Technology Assessment “Treatment Inventory of Costs in Patients with psychiatric disorders” questionnaire (TiC-P) and used for costing from a societal and public healthcare perspective.

For the health-economic evaluation, direct medical costs and costs due to absence from work or reduced efficiency during paid or unpaid work will be assessed at baseline and follow-up using the TiC-P [47, 48]. A list of unit cost prices will be utilised to compute healthcare costs on a per-participant basis [49]. The questionnaire’s test–retest reliability has been shown to be acceptable [48]. Budget impact will be presented as a difference in costs, with a comparison of various uptake levels of therapist-guided iCBT-I across different scenarios, estimating the expected change in healthcare system expenditures after different uptake levels of therapist-guided iCBT-I. The costs of healthcare of various intervention mixes (therapist-guided iCBT-I and face-to-face CBT-I) are compared with the *status quo*.

#### Descriptive and exploratory outcomes

Descriptive and exploratory outcomes will be assessed at baseline and post-intervention, unless otherwise stated.

Perceived benefits and disadvantages of online therapy will be assessed using a 29-item self-developed questionnaire (called the Patient Acceptance and Convenience Inventory). Twenty-seven of these items are answered on a 4-point Likert scale (ranging from 1 to 4 with a total score ranging from 27 to 108) and two are open-ended questions (see Appendix 1 for details). At post-intervention, session attendance will be assessed with one item and adherence to CBT-I methods with nine self-developed items, each rated on a 5-point Likert scale with response options ranging from 1 to 5 and a total score ranging from 9 to 45 (see Appendix 1 for details).

Attitudes towards internet-delivered psychotherapy will be measured employing the subscale “Relative advantage” of the E-Therapy Attitude Measure (ETAM; [31]), consisting of six items, with response options ranging from 1 to 5, a total score from 6 to 30, and excellent internal consistency, with *α* = 0.92.

Attitudes towards internet-delivered as well as face-to-face psychotherapy will additionally be assessed using the “Attitudes” subscales of the e-Therapy Attitudes and Process Questionnaire (eTAP; [50]) and Therapy Attitudes and Process Questionnaire (TAP; [51]). Both subscales have four items, each rated on a 7-point Likert scale (1–7) resulting in total scores ranging from 4 to 28. Internal consistency for these two subscales has been reported to be excellent, with *α* values of 0.92 (eTAP) and 0.91 (TAP), respectively.

Patients’ preference for the therapy format (one item with three response options), how well they can imagine engaging in the therapist-guided iCBT-I as well as face-to-face delivered CBT-I (two items, each rated on a 4-point Likert scale) and most important reasons for or against therapist-guided iCBT-I (two open-ended questions) will be measured using five self-developed items (see Appendix 1 for details).

User experience of the therapist-guided iCBT-I will be measured at post-intervention (only for the iCBT-I group) using the Short Version of the User Experience Questionnaire (UEQ-S; [52]). For this, the reported internal consistencies are good (subscales: Pragmatic Quality: *α* = 0.85; Hedonic Quality: *α* = 0.81).

All self-developed questionnaires (German and English translation) can be found in the Appendices in Additional File 1.

#### Safety

Adverse events related to treatment will be assessed at baseline and post-intervention in two ways. First, the occurrence and impact of possible negative effects during treatment will be measured using the 20-item Negative Effects Questionnaire (NEQ; [53]). The NEQ’s total scores range from 0 to 20 for the occurrence and 0 to 80 for the impact of negative effects. The questionnaire’s internal consistency has been reported to be *α* = 0.95. Second, a self-developed 24-item questionnaire on adverse effects commonly observed during CBT-I will be used (Simon L, Rozental A, Spiegelhalder K, et al: The Negative Effect Questionnaire - Insomnia - An amendment of the Negative Effect Questionnaire for the treatment of insomnia, in preparation).

Furthermore, adverse events related to treatment (e.g. fatigue, sleepiness) will be assessed continuously during treatment. In the therapist-guided iCBT-I group, patients will be asked to report adverse events in sessions 2 through 6. During face-to-face CBT-I, therapists will ask for adverse events and record them in a designated electronic case report form (eCRF). Also, concomitant medication will be recorded in sessions 4 and 6 in both conditions (see Appendix 1 for details).

#### Potential moderators of treatment efficacy

At baseline, demographic variables (e.g. age, gender), the duration of insomnia, and concomitant use of sleep medication will be documented as potential moderators of treatment efficacy. As further potential moderators, the following variables will be assessed at baseline: (a) patients’ chronotype, measured using the 13-item Composite Scale of Morningness (CSM; [54]; *α* = 0.84 [54] to 0.87 [55]); (b) treatment expectations and evaluations using the short form of the Patient Questionnaire on Therapy Expectation and Evaluation (PATHEV; [56]; *α* = 0.82 (Suitability) to 0.89 (Hope of Improvement), depending on subscale); (c) perceived usefulness of internet-delivered psychotherapy employing the Perceived Usefulness subscale of the ETAM ([31], 2017; *α* = 0.87); (d) patients’ perceptions of control over attending internet-delivered/face-to-face psychotherapy using the Perceived Behaviour Control subscales of the eTAP and TAP [50, 51]; (e) prior experiences with internet-delivered interventions/apps and face-to-face psychotherapy using five self-developed items; (f) attitudes towards treatment expectations using two self-developed items, each rated on a 4-point Likert scale; and (g) the degree to which patients feel informed about the characteristics of both intervention arms, utilising four self-developed items, each rated on a 4-point Likert scale (see Appendix 1 for further details about e, f, and g).

Moreover, therapists will briefly assess patients’ adherence to CBT-I after each session. They will also document the patient’s agreed bedtime window, which will be used to calculate adherence to bedtime restriction [57]. Finally, for health-economic analyses, therapists will document the time that they spent on each patient’s therapy (see Appendix 2 for details).

### Patient timeline {13}

Figure 1 shows the patient timeline.

### Sample size {14}

Since there is neither a validated non-inferiority margin for the insomnia treatment nor an established procedure for determining one, we followed the recommendation of the Delta2 group and considered different perspectives, including anchor-based clinical judgement and distribution-based reasoning to select the non-inferiority margin of the primary outcome (ISI) for this trial [58]. First, most clinicians involved in this trial agreed that new insomnia treatments are not inferior to the first-line treatment if the difference in post-intervention ISI scores is less than 2 points. Accordingly, 2 or more points were considered the minimally important difference (ISI range 0–28 points). From a distribution-based perspective, *d* = 0.5 is a commonly used lower limit for the smallest difference considered important [59], which corresponds to 2.3 points on the ISI. Second, as the non-inferiority margin should be smaller than the minimal important difference, 2 points on the ISI are an upper limit for the non-inferiority margin. Contrary to the minimal important difference, the non-inferiority margin should describe a clinically negligible difference. We agreed that the boundary between “minimal important” and “clinically negligible” is not sharply delineated, meaning that the boundary should not be set directly; for example, 0.1 points below the clinically relevant difference. There was no doubt that a 1-point difference is a clinically negligible difference. From a distribution-based perspective, 0.9 points correspond to a *d* = 0.2 (small) effect size according to Cohen’s taxonomy. Third, integrating these different perspectives, clinical and statistical arguments, with clinical reasoning being more important to us, a group difference δ of 1.5 points was conservatively considered clinically negligible and hence chosen as the non-inferiority margin. We intend to demonstrate non-inferiority of therapist-guided iCBT-I versus face-to-face CBT-I with regard to post-intervention ISI scores based on a shifted *t*-test at a one-sided significance level of 2.5%. To test for non-inferiority, the null hypothesis that ISI scores in the face-to-face CBT-I arm are ≥ 1.5 points higher than in the therapist-guided iCBT-I arm with an assumed common standard deviation of 4.5 points is rejected if the two-sided 95% confidence interval (CI) for the difference in ISI scores (control versus experimental) is < 1.5 points. To achieve 90% power, 382 patients must be included in analysis (191 per group; nQuery Advisor 7.0). Non-compliance and drop-out after randomisation are assumed to be no more than 10% [37]. Therefore, 422 patients will be randomised.

### Recruitment {15}

Several recruitment strategies are in place for achieving adequate patient enrolment. First, participating centres will recruit patients from their outpatient facilities. Second, press releases and established contacts with major German online and print media will be used to announce the trial. Interested subjects may visit the project website and provide their ZIP code. They will then be referred to the nearest study centre within 1-h travel distance of the patients’ residence, if possible. Third, recruitment will be supported by the German Sleep Society (DGSM) and the section Sleep Medicine of the German Association for Psychiatry, Psychotherapy and Psychosomatics (DGPPN).

## Assignment of interventions: allocation

### Sequence generation {16a}

Patients will be randomised to one of the two treatment arms with a 1:1 allocation ratio. Treatment allocation will be done centrally in the eCRF in REDCap® by the Clinical Trials Center (CTU) of the Medical Center—University of Freiburg. For each study centre, block randomisation with varying block sizes will be performed, with all group allocations concealed from the investigators to minimise selection bias.

### Concealment mechanism {16b}

The allocation sequence will be concealed until interventions are assigned as therapists randomise patients in the eCRF by clicking an icon.

### Implementation {16c}

The allocation sequence will be created centrally in the eCRF using the web-based data entry system REDCap® by the Clinical Trials Center (CTU) of the University of Freiburg Medical Center. Therapists at the study centres will enrol patients and assign them to the interventions by clicking on the “randomise” icon in the eCRF.

## Assignment of interventions: blinding

### Who will be blinded {17a}

Blinding of patients and therapists is impossible due to the specific characteristics of the interventions. Participating patients will be thoroughly informed about the study; hence, they will understand the treatment arm to which they have been assigned. Data analysts will not be blinded.

### Procedure for unblinding if needed {17b}

Not applicable due to the lack of blinding.

## Data collection and management

### Plans for assessment and collection of outcomes {18a}

Patient data will be collected in pseudonymised form with an electronic Case Report Form (eCRF) using REDCap®. Access to the eCRF will be granted to authorised study therapists and further members of the study team only, and only if they have received appropriate training in working with the system. In addition, a comprehensive manual will be provided. Study therapists will add new patients and document patients’ baseline data and further CBT-I session data in the eCRF. Data will be verified during data entry by built-in format and edit checks and so-called “data quality rules”. Patients will be invited automatically to online surveys to answer baseline, post-intervention, and follow-up questionnaires. In addition, sleep diary data will be collected. In the internet-delivered treatment, this will be done through a progressive, access-controlled web app. In the face-to-face treatment, patients will receive a folder containing a sleep diary in paper form. After completing the treatment, the sleep diary will be sent to the coordinating centre at the University of Freiburg Medical Center. Patient identification is ensured only by the study-specific pseudonym, not by the person’s name or any other personal data. Study team members will transfer the data into the eCRF. Details on the specific assessments and questionnaires can be found in Fig. 1 and in section “Outcomes {12}”.

### Plans to promote patient retention and complete follow-up {18b}

During recruitment, patients will receive detailed information about the requirements of study participation. To achieve high compliance, they will be randomised after completing the baseline questionnaire and directly before being able to participate in the intervention. Patients may discontinue participation in the intervention at any time without providing a reason or suffering any negative consequences. All study patients, including those who discontinue treatment, will be contacted via email and asked to complete the post-intervention and follow-up assessments. All questionnaires at all measurement points will be filled out using an online survey, providing a high level of flexibility and convenience for patients. Reminders for filling out the questionnaires will be sent automatically to individual patients who have not yet completed the questionnaires. To promote patient retention, we will employ the strategies for increasing response rates to questionnaires recommended in the Cochrane review conducted by Edwards et al. [60]. Given that a trial evaluating a previous version of the therapist-guided iCBT-I intervention showed a drop-out rate of 4.6% in the intervention group [37], non-compliance and drop-out are assumed to be no more than 10% in this trial. In the case of protocol deviations, as many outcomes as possible will be collected.

### Data management {19}

The setup of the eCRF and data management will be performed with REDCap®, which is developed and maintained by the REDCap® Consortium (redcap@vanderbilt.edu). This system uses built-in security features to prevent unauthorised access to patient data, including an encrypted transport protocol for data transmission from the participating study centres to the study database. An audit trail provides a history of the data entered, changed, or deleted, indicating the processor and date. The study database is located on a server of the information technology facility (Zentrum für Digitalisierung und Informationstechnologie, ZDI) at University of Freiburg Medical Center. Employees of the Clinical Trials Unit charged with hosting the eCRF and the study database will be obliged to maintain data confidentiality and to comply with data-protection regulations. Access will be granted to authorised personnel only. Technical specifications of the database will be described in the codebook delivered automatically by the REDCap® system.

Before entering any data, the trial database and eCRFs will be validated. Study therapists and further members of the study team will not be granted access to the eCRF until they have been trained and have signed an access form. Data will be verified during data entry by built-in format and edit checks and data quality rules.

### Confidentiality {27}

All research data will be stored using a unique study identification code for each patient. The reference lists that link personal data with the study identification code will only be accessible to the respective participating study centres and securely stored on site. These lists will be deleted 10 years after data collection is complete (after the last patient has completed the final survey), by employees delegated for this purpose in a two-person review (= good clinical practice (GCP)-compliant). This timely deletion will be ensured by the study centres. Scientific evaluations will be carried out in a pseudonymised manner, and the results will not allow conclusions to be drawn about the patients’ identity. In scientific publications, study results will be presented in an anonymised format, ensuring that making any inferences about individuals is impossible.

### Plans for collection, laboratory evaluation and storage of biological specimens for genetic or molecular analysis in this trial/future use {33}

Not applicable because no biological specimens will be collected.

## Statistical methods

### Statistical methods for primary and secondary outcomes {20a}

#### Efficacy

The primary efficacy analysis will be performed in the per-protocol set, excluding patients with major protocol violations since it is generally recognised that “in an equivalence or non-inferiority trial, use of the full analysis set is generally not conservative” (European Medicines Agency—CPMP/ICH/363/96, Sect. 5.2.3, p. 26). For sensitivity analysis, the same analysis will be performed according to the intention-to-treat principle, based on the full analysis set, including all randomised patients with their original treatment allocation. The effects of internet-delivered and face-to-face CBT-I—with respect to the primary endpoint ISI at post-intervention—will be tested within a linear mixed model for repeated measures with corresponding two-sided 95% confidence intervals (CIs). The model will include treatment and study centre as independent variables. In cases involving low recruitment rates at specific centres, additional sensitivity analyses will be performed. The one-sided test of non-inferiority (internet-delivered versus face-to-face CBT-I) at a significance level of 2.5% will be based on the two-sided 95% CI from the mixed model for repeated measures. The null hypothesis will be rejected if the lower limit of the CI for the difference lies entirely above -1.5 points.

If non-inferiority is concluded, a test for superiority of therapist-guided iCBT-I versus face-to-face CBT-I will be conducted. For this, the primary analysis will be repeated in the full analysis set. If the resulting two-sided 95% CI lies entirely below zero, superiority of therapist-guided iCBT-I versus face-to-face CBT-I will be concluded. As this is a closed test procedure, it will not be necessary to adjust the significance level *α* (see “Points to consider on switching between superiority and non-inferiority” EMA—CPMP/EWP/482/99). Analysis in the mixed model for repeated measures will permit valid inferences under the “missing at random” assumption, which seems justified since we expect that most of the missing data will be absent for reasons probably unrelated to the missing outcomes. Secondary endpoints will be analysed in a fashion similar to the primary outcome, using regression models, as appropriate, for the respective type of data. Treatment effects will be calculated using two-sided 95% CI.

#### Economic evaluation

The health-economic evaluation will be performed from a societal and a public healthcare perspective with a time horizon of 6 months. In the full analysis set, we will employ two multilevel models, one to assess costs and the other to examine effects, taking into account the data’s hierarchical structure. For effects, normal-based 95% CIs will be estimated whereas for costs, 95% CIs will be estimated using bias-corrected and accelerated bootstrapping with 5000 replications [61]. Multilevel models will be combined with cluster bootstrapping [62], wherein entire clusters rather than individual data points will be resampled, thereby preserving the hierarchical data structure. The bootstrapped cost and effect pairs will be presented on both (a) a cost-effectiveness plane and (b) a cost-effective acceptability curve disclosing the probability that therapist-guided iCBT-I is cost-effective for a wide range of willingness-to-pay thresholds [63]. When the health outcomes produced are empirically proven to be equivalent, a cost-minimization analysis (CMA) will be deemed an appropriate methodology [64]. Sensitivity analyses will be performed to assess the impact of the varying input parameters used in the analyses.

#### Budget impact analysis

A budget impact analysis will be performed to evaluate the impact of the implementation of therapist-guided iCBT-I on the German healthcare system. This budget impact analysis will be performed in accordance with the International Society for Pharmacoeconomics and Outcomes Research guidelines [65]. The analysis will adopt a healthcare perspective. The analytical decision model will use a time horizon of 5 years to capture the long-term health effects of therapist-guided iCBT-I without excessively extrapolating from the available evidence. We will compare different scenarios, examining varying levels of implementation of therapist-guided iCBT-I. Sensitivity analyses will be conducted to assess the robustness of the results.

### Interim analyses {21b}

There are no interim analyses planned.

### Methods for additional analyses (e.g. subgroup analyses) {20b}

Exploratory moderator analyses will be used to investigate whether pre-treatment patient characteristics are associated with differential treatment efficacy. Potential moderators include clinical (e.g. insomnia severity) and treatment-related variables (e.g. attitudes towards internet-delivered treatments or preference for a therapy format; see section “Outcomes {12}”, under “Descriptive and exploratory outcomes”). For each patient, a personalised multivariate model will be constructed predicting the differential efficacy of internet-delivered versus face-to-face treatment.

### Methods in analysis to handle protocol non-adherence and any statistical methods to handle missing data {20c}

Data—especially data on the clinical outcome—should be collected regardless of the patient’s adherence to the protocol to obtain the best approximation to the full analysis set. Data should also be collected on other therapies received after study drop-out. Specifically, full details should be collected about the type of additional (non-randomised) therapy used, including when and for how long it was used and at what dose. We will describe the frequency and type of protocol violations and missing values. Graphical summaries (Kaplan–Meier plots) of the drop-out patterns will be provided to investigate the drop-out pattern between treatment groups. In addition, we will investigate whether there are imbalances in relevant factors with respect to missing values and whether patients with and without missing values have different characteristics at baseline.

### Plans to give access to the full protocol, patient-level data, and statistical code {31c}

After completion of the clinical study and upon reasonable request, the anonymised patient-level data may be shared with third parties exclusively for scientific purposes (e.g. research questions requiring special analytical capabilities, combining multiple datasets for meta-analyses, re-analysing study results by independent research institutions to ensure good scientific practice).

## Oversight and monitoring

### Composition of the coordinating centre and trial steering committee {5d}

There are monthly meetings in which the principal investigators, the study coordinator, and all collaborating partners meet online to discuss the current study status. In addition, there is a weekly meeting between the Freiburg and Lüneburg study teams. The Freiburg study team is primarily responsible for the face-to-face arm, providing training on CBT-I techniques, and implementing face-to-face therapy in this trial. The Lüneburg study team is primarily responsible for the online arm and providing training on using the online therapy programme and sleep diary app. Potential study participants and enrolled study patients can contact the Freiburg study team via email or phone on a study hotline for general study-related questions. For technical difficulties with the online therapy programme or the sleep diary app, support via email will be provided by the Lüneburg study team.

Clinical monitoring will also be implemented. The clinical monitors are part of the Freiburg study team and will monitor the current trial to ensure the highest quality of data and effective communication with the participating centres. All participating centres have agreed that these researchers are allowed to visit the centres before, during, and after completion of the study to ensure that the study is conducted, recorded, and reported according to the study protocol, relevant standard operating procedures, and requirements of the ICH-GCP (International Conference on Harmonization – Good Clinical Practice). As part of the initiation visit, the study will be described in detail, the Investigator Site File (ISF) discussed, and eCRF training conducted. In addition, a minimum of two interim visits is planned for each centre. The first visit is scheduled to take place shortly after the start of recruitment, allowing for the early correction of any potential systematic errors. Depending on the experience level of the centres, additional visits and greater support might be required. Generally, the level of study experience influences the training intensity during initiation and the level of support provided (risk-adapted approach). Between visits, the monitors will regularly inquire about study progress at the centres and offer support via telephone. Finally, there will be one close-out visit at the trial’s end. To ensure high data quality, 10% of face-to-face CBT-I sessions will be videotaped and checked by independent raters for adherence to the manual. Likewise, 10% of the cases treated with therapist-guided iCBT-I will be checked. If therapists are discovered to have deviated from the treatment manual, they will be offered additional support.

In addition, we have set up a Scientific Advisory Board that will assume responsibility for the scientific integrity of the clinical trial, scientific validity of the study protocol, and scientific quality of the final study report.

### Composition of the data monitoring committee, its role and reporting structure {21a}

The data monitoring committee (DMC) is an independent multidisciplinary group consisting of one biostatistician and two clinicians that, collectively, have experience in the management of patients with insomnia disorder and the conduct and monitoring of RCTs. The DMC is independent from the sponsor and has no competing interests. It will be responsible for safeguarding the interests of trial patients, assessing the safety of the interventions used during the trial, and monitoring the overall conduct of the clinical trial. Three meetings of the DMC and the investigators will be scheduled to evaluate (a) any safety issues related to the trial, (b) potential protocol violations, and (c) recruitment rates. Each meeting will consist of an open session (attended by the principal investigators, the study statistician, and the DMC) and a closed session at the end (restricted to the DMC members). The first meeting will take place after the enrolment of 40 patients, the second after enrolment of 150 patients, and the third after enrolment of 350 patients. If patient recruitment is slower than expected, the DMC will be informed and a meeting scheduled earlier than initially planned. Data to be reviewed regarding safety and study conduct include patient recruitment and withdrawal information, adverse events, and a summary of protocol violations that were documented in the eCRF. The DMC will provide recommendations to the investigators concerning any continuation or termination of, or other modifications to the study.

### Adverse event reporting and harms {22}

CBT-I is a well-studied treatment that is recommended by current clinical guidelines (e.g. [15]). The most common adverse events of CBT-I are transient sleepiness or fatigue during the initial treatment phase. There is no evidence that serious adverse events or harms should be expected. However, adverse events related to treatment will be assessed continuously during treatment. Furthermore, adverse events will be assessed at baseline and post-intervention using two questionnaires (for details, see “Safety” under “Outcomes”).

### Frequency and plans for auditing trial conduct {23}

No clinical trial audit is planned. Nevertheless, the German Research Foundation (Deutsche Forschungsgemeinschaft; DFG) as well as the regional council reserve the right to conduct a study audit. If there is an audit, the process will be independent of the investigators and sponsor.

### Plans for communicating important protocol amendments to relevant parties (e.g. trial patients, ethical committees) {25}

If protocol amendments are made, these will be submitted to the responsible ethics committee(s). In addition, all study centres will be informed immediately.

### Dissemination plans {31a}

Regardless of the study’s outcomes, its results will be published in international peer-reviewed journals. A project website (https://www.isleep-well.de) informs the public (including policymakers and the media) about the aims and main outcomes of this trial. The scientific results of the consortium will be communicated at scientific meetings and conferences and by publication in peer-reviewed journals. All scientific publications will follow the open access policy of the DFG. In addition, patient groups will be contacted to inform both their members and the wider audience about the trial’s results.

## Discussion

### Study objectives

This study protocol describes a randomised controlled non-inferiority trial investigating the efficacy of therapist-guided iCBT-I versus face-to-face CBT-I. It is assumed that both treatment formats will lead to a reduction in patient-reported insomnia severity, with clinically negligible differences between these two formats. This finding would be important, given that insomnia disorder is highly prevalent and associated with several negative outcomes for individuals and society. Although CBT-I is recommended as first-line treatment by clinical guidelines, only a small minority of patients have access to this therapy. Thus, demonstrating the non-inferiority of therapist-guided iCBT-I has the potential for treatment to reach a broader patient population and, thereby, contribute to wider dissemination of first-line treatment of insomnia.

### Study contributions

The evidence on the comparative efficacy of face-to-face CBT-I and therapist-guided iCBT-I is inconclusive, and clinical recommendations lack evidence, which is why the planned study can contribute in several ways.

First, to the best of our knowledge, it will be the first time that individual face-to-face therapy is compared with therapist-guided iCBT-I in a planned non-inferiority study. Therefore, if face-to-face CBT-I is superior, it cannot be argued that lacking support from a therapist explains the smaller effects of therapist-guided iCBT-I. Moreover, different levels of therapist qualifications between treatment conditions were discussed as a limiting factor in prior studies. To avoid this limitation in the current study, all therapists will provide both treatment conditions. In addition, therapists’ adherence to each therapy will be assessed by having an independent rater analyse 10% of the sessions.

Second, the non-inferiority margin in the present study is considerably smaller than that employed by Blom et al. [23] and slightly smaller than that employed by Kallestadt et al. [25]. It must be acknowledged that there is a lack of studies validating margins for insomnia, and specifying certain margins could be criticised as arbitrary. The comparatively small margin in the present trial leads to a considerably larger sample size and the need to invest more financial and human resources to conduct the study. However, from a patient safety perspective, this might be preferred, as the risk of falsely claiming clinically neglectable differences is reduced. For non-inferiority trials, using per-protocol samples is recommended as the more conservative approach [66]. Although per-protocol analyses were reported in prior trials [23, 25], they used intention-to-treat samples for the primary analyses. More importantly, contrary to the current study, those trials were not powered for per-protocol samples. The present study will provide data on 422 patients, adding significantly to the results of the total of 267 patients spanning all prior published studies on this topic [23–26].

Third, one previous study found that patients preferred face-to-face CBT-I before receiving this treatment, and patients receiving face-to-face CBT-I were more satisfied afterwards than patients receiving therapist-guided iCBT-I [24]. The present study will contribute to these findings by evaluating patients’ attitudes towards internet-delivered and face-to-face treatment, including treatment preferences, expectations, and acceptance, including the perceived convenience of each treatment format. Furthermore, these characteristics will be assessed before and after treatment to explore whether personal experiences with the respective therapy alter patients’ attitudes and expectations. In addition, advocates of internet-delivered therapy regularly highlight potential benefits, such as time and location independence or anonymous usability [18]. However, for CBT-I, it is largely unknown whether these advantages are actually experienced by patients. The comprehensive assessment of potential benefits will provide insights from a patient’s perspective.

Fourth, practitioners might hesitate to recommend digital therapy due to patient safety concerns. As prior trials focused on desirable outcomes, these concerns were only considered in a few studies [23, 26]. The present study will systematically investigate negative side effects and adverse events post-treatment as well as throughout treatment, thereby providing more data on the safety of therapist-guided iCBT-I.

Fifth, health-economic evaluations are particularly important for policymakers to have evidence for the best possible allocation of limited resources within the healthcare system [64]. While prior health-economic evaluations of therapist-guided iCBT-I (e.g. [47]) have demonstrated cost-effectiveness when compared to patients being on a waiting list, the present study will provide data for two effective treatments for the first time.

Finally, most participating centres are specialised in sleep medicine and provide CBT-I in routine care. Therefore, the current study serves as a template on how to integrate internet-delivered CBT-I into routine care processes, thereby facilitating later implementation.

### Limitations

Despite the potential strengths and contributions of the present study, several limitations must be considered.

First, as face-to-face is the standard format in which CBT-I is offered, it is expected that therapists are more experienced in providing face-to-face therapy than communicating with patients in written form, which is part of therapist-guided iCBT-I. This might lower the efficacy of therapist-guided iCBT-I. To mitigate this potential bias, all therapists will receive a manual on therapist-guided iCBT-I, including extensive information about providing written feedback as well as templates and boilerplates. Moreover, they must engage in two consecutive workshops in which they are trained to provide written feedback to patients receiving therapist-guided iCBT-I.

Second, it is assumed that internet-delivered therapy saves therapists’ time, as standard educational parts of therapy are provided by the online platform. However, this potential economic benefit may be neutralised or even reversed, since therapists who lack experience in offering guidance within the therapist-guided iCBT-I might need more time to provide written feedback, thereby making internet-delivered therapy more time-consuming and, hence, expensive. Against this background, therapists will be instructed to restrict their time to 60 min per session per patient for providing feedback. The study will assess the weekly time needed for each of the two CBT-I approaches, allowing direct comparison of the time required by therapists.

Third, the economic advantages of therapist-guided iCBT-I might be more likely when using self-help instead of therapist-guided iCBT-I. However, therapist-guided iCBT-I seems to be more accepted by patients [29] and likely more effective [29, 30]. Moreover, there is no evidence that an unguided programme is more effective than therapist-guided digital therapy. Therefore, a conservative approach was applied by choosing this variant of therapist-guided iCBT-I, which appears to have the highest likelihood of being non-inferior to face-to-face CBT-I.

Fourth, the internet-delivered CBT-I programme in the present study is based on an already-evaluated programme for workers [37]; but the current version (in which job-related references were removed) is being used for the first time. Although this intervention was pre-tested with help-seekers, unforeseen technical or conceptual issues might arise in a larger group of users, which might negatively affect user experience and limit efficacy. In cases of technical issues, our assessment of user experience will allow us to quantify this unintended effect, as user experience has been found to be associated with efficacy [67].

## Conclusions

Evidence in the current literature supporting the non-inferiority of therapist-guided iCBT-I is scarce and yields inconclusive findings. Prior studies also suffer from methodological limitations, such as small sample sizes or comparators not representative of routine care. This randomised controlled trial will (a) investigate the non-inferiority of therapist-guided iCBT-I versus face-to-face CBT-I with regard to insomnia severity at post-intervention in an adequately powered sample and with the same therapeutic content and same level of therapist qualification provided to both treatment groups; (b) conduct a health-economic evaluation; and (c) explore potential benefits and disadvantages of therapist-guided iCBT-I. If this non-inferiority trial demonstrates that therapist-guided iCBT-I is a fully viable alternative to face-to-face CBT-I, policymakers may be encouraged to implement therapist-guided iCBT-I in routine care, and healthcare providers might be more inclined to recommend this treatment to their patients.

## Trial status

Patient recruitment started in June 2023. The current protocol is version 4 of 28–2-2023. Presently (26 February 2024), we have enrolled 106 patients. Patient recruitment is estimated to be completed by roughly December 2024.

### Supplementary Information

Supplementary Material 1.

## Data Availability

Self-developed questionnaires are available in the Appendix (see Additional File 1). After completion of the clinical study, the data will be anonymized, and if necessary, these anonymized data may be shared with third parties exclusively for scientific purposes (e.g. questions requiring special analytical capabilities, combining multiple datasets for meta-analyses, re-analysing study results by independent research institutions to ensure good scientific practice).

## Abbreviations

AQoL-6D: Assessment of Quality of Life
AUC: Area under the curve
CBT-I: Cognitive behavioural therapy for insomnia
CMA: Cost-minimization analysis
CSM: Composite Scale of Morningness
DGPPN: German Association for Psychiatry, Psychotherapy and Psychosomatics
DGSM: German Sleep Society
DMC: Data monitoring committee
eCRF: Electronic case report form
EQ-5D-5L: European Quality of Life 5 Dimensions 5 Level Versions
ETAM: E-Therapy Attitude Measure
eTAP: e-Therapy Attitudes and Process Questionnaire
FSS: Fatigue Severity Scale
GCP: Good clinical practice
iCBT-I: Internet-delivered cognitive behavioural therapy for insomnia
IPAQ-SF: International Physical Activity Questionnaire - Short Form
ISF: Investigator Site File
ISI: Insomnia Severity Index
NEQ: Negative Effects Questionnaire
PATHEV: Patient Questionnaire on Therapy Expectation and Evaluation
PHQ-9: Patient Health Questionnaire
MET: Metabolic equivalent
QALYs: Quality-adjusted life years
TAP: Therapy Attitudes and Process Questionnaire
TiC-P: Treatment Inventory of Costs in Patients with psychiatric disorders
UEQ-S: User Experience Questionnaire - Short Version
ZDI: Zentrum für Digitalisierung und Informationstechnologie of Medical Center - University of Freiburg
CTU: Clinical Trials Center
