# Supplementary material for: Investigating non-inferiority of internet-delivered versus face-to-face cognitive behavioural therapy for insomnia (CBT-I): a randomised controlled trial (iSleep well)
Source: Trials. 2024 Jun 10;25:371. doi: 10.1186/s13063-024-08214-6 (PMC11163861; doi:10.1186/s13063-024-08214-6)
Supplement: Supplementary file 1 — Supplementary Material 1. [file 13063_2024_8214_MOESM1_ESM.pdf]

Additional File 1

Supplementary Material for:
Benz, F., Grolig, L., Hannibal, S., Buntrock, C., Cuijpers, P., Domschke, K., Ebert, D.D., Janneck, M., Jenkner, C., Johann, A.F., Josef, A., Kaufmann, M., Koß, A., Mallwitz, T., Mergan, H., Morin, C. M., Riemann, D., Riper, H., Schmid, S. R., Smit, F., Spille, L., Steinmetz, L., van Someren, E.J.W., Lehr, D. & Spiegelhalder, K. (2024). Investigating non-inferiority of internet-delivered versus face-to-face cognitive behavioural therapy for insomnia (CBT-I): a randomised controlled trial (iSleep well).

Note. All items were translated into English by LG and a graduate student of psychology (Natalie Slawik), and then back translated into German by a bilingual health psychology researcher (Patricia Nixon). Differences between original and back translated items were resolved by discussion (FB, LG, LSp.).

Appendix 1: Self-developed questionnaire items for patients

Original Items (German): Baseline	Translated Items (English): Baseline
**Erfahrung mit Online-Therapieprogrammen und Apps**<br><br>1. Haben Sie schon einmal Online-Therapieprogramme oder Apps zur Bewältigung von Schlafbeschwerden in Anspruch genommen?<br>___ *Ja, eins*<br>___ *Ja, mehrere*<br>___ *Nein*<br><br>Bei „Ja, eins“ folgende Fragen 1x, bei „Ja, mehrere“ Mehrfachnennungen hintereinander ermöglichen (und Text: „Bitte nennen Sie die Programme oder Apps einzeln nacheinander“):<br><br>Welche(s) war das? _______ *(freies Textfeld)*<br>War das ein begleitetes Format (mit persönlichen Rückmeldungen durch eine/n Coach oder Therapeutin/Therapeut, z. B. via E-Mail, SMS, Chat oder Telefon) oder ein Selbsthilfeformat?<br>*o Begleitetes Format*<br>*o Selbsthilfeformat*<br><br>2. Haben Sie schon einmal Online-Therapieprogramme oder Apps zur Bewältigung einer anderen psychischen Erkrankung in Anspruch genommen?<br>___ *Ja, eins*<br>___ *Ja, mehrere*<br>___ *Nein*<br><br>Bei „Ja, eins“ folgende Fragen 1x, bei „Ja, mehrere“ Mehrfachnennungen hintereinander ermöglichen (und Text ergänzen: „Bitte nennen Sie die Programme oder Apps einzeln	**Experience with online therapy programmes and apps**<br><br>1. Have you ever used online therapy programmes or apps to cope with sleep problems?<br>___ *Yes, one*<br>___ *Yes, several*<br>___ *No*<br><br>If "Yes, one" ask the following questions 1x; if "Yes, several" allow multiple responses in a row (and add text: "Please name the programmes or apps one by one"):<br><br>Which one(s)? _______ *(free text field)*<br>Was this a guided format (with personal feedback from a coach or therapist, e.g., via email, text message, chat, or phone) or a self-help format?<br>*o Guided format*<br>*o Self-help format*<br><br>2. Have you ever used online therapy programmes or apps to cope with another mental illness?<br>___ *Yes, one*<br>___ *Yes, several*<br>___ *No*<br><br>If "Yes, one" ask the following questions 1x; if "Yes, several" allow multiple responses in a row (and add text: "Please name the programmes or apps

nacheinander“):

Welche(s) war das? _______ *(freies Textfeld)*
Wofür? ______ *(freies Textfeld)*
War das ein begleitetes Format (mit persönlichen Rückmeldungen durch Coach oder Therapeutin, z. B. via E-Mail, SMS, Chat oder Telefon) oder ein Selbsthilfeformat?
o Begleitetes Format
o Selbsthilfeformat

3. Haben Sie schon einmal Online-Programme oder Apps zur allgemeinen Gesundheitsförderung (z.B. Stressbewältigungstraining, Entspannungstraining, Ernährungstraining, Gesundheitsapp) in Anspruch genommen?
___ *Ja, eins*
___ *Ja, mehrere*
___ *Nein*

Bei „Ja, eins“ folgende Fragen 1x, bei „Ja, mehrere“ Mehrfachnennungen hintereinander ermöglichen (Und Text ergänzen: „Bitte nennen Sie die Programme oder Apps einzeln nacheinander“):

Welche(s) war das? _______ *(freies Textfeld)*
Wofür? _____ *(freies Textfeld)*

War das ein begleitetes Format (mit persönlichen Rückmeldungen durch Coach oder Therapeutin, z. B. via E-Mail, SMS, Chat oder Telefon) oder ein Selbsthilfeformat?
o Begleitetes Format
o Selbsthilfeformat

Erfahrung mit Psychotherapie vor Ort

1. Haben Sie schon einmal eine ambulante Psychotherapie in Anspruch genommen?
___ *Ja* ___ *Nein*

Wenn Ja:

1.1 Wie viele ambulante Psychotherapien haben Sie bislang in Anspruch genommen?
_______ (Matrix: 1. Anzahl Therapien
2. Wie lange hat die Therapie gedauert? Mehrfachauswahl: *weniger als 1 Jahr; 1-2 Jahre; 2-3 Jahre; länger als 3 Jahre*)

1.2 Wann hat Ihre letzte Psychotherapie geendet?
_______ *(freies Textfeld)*

one by one"):

Which one(s)? _______ *(free text field)*
For what? ______*(free text field)*
Was this a guided format (with personal feedback from a coach or therapist, e.g., via mail, text message, chat, or phone) or a self-help format?
o Guided format
o Self-help format

3. Have you ever used online programmes or apps for general health promotion (e. g., stress management training, relaxation training, nutrition training, health app)?
___ *Yes, one*
___ *Yes, several*
___ *No*

If "Yes, one" ask the following questions 1x; if "Yes, several" allow multiple responses in a row (and add text: "Please name the programmes or apps one by one"):

Which one(s)? _______*(free text field)*
For what? ______*(free text field)*

Was this a guided format (with personal feedback from coach or therapist, e.g., via mail, text message, chat, or phone) or a self-help format?
o Guided format
o Self-help format

Experience with on-site psychotherapy

1. Have you ever received outpatient psychotherapy?
___ *Yes* ___ *No*

If Yes:

1.1 How many outpatient psychotherapies have you received to date?
_______ (Matrix: 1. number of therapies
2. How long did the therapy last? Multiple choice: *less than 1 year; 1-2 years; 2-3 years; longer than 3 years*).

1.2 When did your last psychotherapy end?
_______ *(free text field)*

1.3 Um welches Format handelte es sich dabei? (Mehrfachnennungen möglich)<br>*o Psychotherapie vor Ort*<br>*o Psychotherapie via Video*<br>*o Sonstiges, und zwar _______ (freies Textfeld)*<br><br>1.4 Wofür haben Sie die Psychotherapie in Anspruch genommen? (Mehrfachnennungen möglich)<br>*o Zur Behandlung von Schlafstörungen*<br>*o Zur Behandlung einer Depression*<br>*o Zur Behandlung von Ängsten*<br>*o Zur Behandlung von Sucht*<br>*o Zur Behandlung einer anderen psychischen Erkrankung, und zwar: _______ (freies Textfeld)*<br><br>2. Haben Sie schon einmal eine tagesklinische oder stationäre Behandlung in einer Klinik für Psychiatrie oder Psychosomatik in Anspruch genommen?<br>___ *Ja* ___ *Nein*<br><br>2.1 Wie viele tagesklinische oder stationäre Behandlungen haben Sie bislang in Anspruch genommen?<br><br>Anzahl Behandlungen _______ *(freies Textfeld)*<br><br>(pro Behandlung) Wie lange hat die tagesklinische oder stationäre Behandlung gedauert? (Mehrfachauswahl)<br>*o bis 3 Wochen*<br>*o 3-6 Wochen*<br>*o 6-12 Wochen*<br>*o länger als 12 Wochen*<br><br>2.2 Wann hat Ihre letzte tagesklinische oder stationäre Behandlung geendet?<br>_______ *(freies Textfeld)*<br><br>2.3 Wofür haben Sie diese tagesklinische oder stationäre Behandlung in Anspruch genommen? (Mehrfachauswahl)<br>o *Zur Behandlung einer primären Insomnie, also chronischen Schlafbeschwerden*<br>*o Zur Behandlung einer Depression*<br>*o Zur Behandlung von Ängsten*<br>*o Zur Behandlung von Sucht*<br>*o Zur Behandlung einer anderen psychischen Erkrankung, und zwar: _______ (freies Textfeld)*	1.3 What kind of format was it in? (multiple answers possible)<br>*o On-site psychotherapy*<br>*o Psychotherapy via video*<br>*o Other, namely _______ (free text field)*<br><br>1.4 What did you seek psychotherapy for? (multiple answers possible)<br>*o For the treatment of sleep disorders*<br>*o For the treatment of depression*<br>*o For the treatment of anxiety*<br>*o For the treatment of addiction*<br>*o For the treatment of another mental illness, namely: _______ (free text field)*<br><br>2. Have you ever received day-care clinic or inpatient treatment in a psychiatric or psychosomatic clinic?<br>___ *Yes* ___ *No*<br><br>2.1 How many day-care or inpatient treatments have you received so far?<br><br>Number of treatments _______ *(free text field)*<br><br>(per treatment) How long did the day-care or inpatient treatment last? (Multiple choice)<br>*o up to 3 weeks*<br>*o 3-6 weeks*<br>*o 6-12 weeks*<br>*o longer than 12 weeks*<br><br>2.2 When did your last day-care or inpatient treatment end?<br>_______ *(free text field)*<br><br>2.3 What did you seek this day-care or inpatient treatment for? (Multiple choice)<br>*o For the treatment of primary insomnia, i.e. chronic sleep problems*<br>*o For the treatment of depression*<br>*o For the treatment of anxiety*<br>*o For the treatment of addiction*<br>*o For the treatment of another mental illness, namely: _______ (free text field)*

**Einstellung zur Behandlungserfolgserwartung**<br><br>Bitte kreuzen Sie nachfolgend an, inwiefern Sie glauben, dass Ihnen die beiden Behandlungsformate (Online-Therapieprogramm mit persönlicher Begleitung durch eine Therapeutin/einen Therapeuten oder Psychotherapie vor Ort) gegen Ihre Schlafbeschwerden helfen können.<br><br>Antwortmöglichkeiten für alle Items: *Trifft nicht zu / Trifft eher nicht zu / Trifft eher zu / Trifft zu*<br><br>1. Ich bin zuversichtlich, dass das Online-Therapieprogramm "iSleep well" mir gut gegen meine Schlafbeschwerden helfen kann.<br>2. Ich bin zuversichtlich, dass die Psychotherapie vor Ort in dieser Studie mir gut gegen meine Schlafbeschwerden helfen kann.	**Attitude towards the expectation of treatment success**<br><br>Please tick below to what extent you believe that the two treatment formats (online therapy programme with personal guidance from a therapist or on-site psychotherapy) can help you with your sleep problems.<br><br>Response options for all items: *Do not agree / Do rather disagree / Do rather agree / Do agree*<br><br>1. I am confident that the online therapy programme "iSleep well" can help me effectively with my sleep problems.<br>2. I am confident that on-site psychotherapy in this study can help me effectively with my sleep problems.
**Wissen über beide Therapie-Formate**<br><br>Jetzt geht es um die Rahmenbedingungen und Inhalte der beiden Therapie-Formate, über die Sie im Vorgespräch und durch die Studienunterlagen informiert wurden. Bitte kreuzen Sie an, inwiefern Sie den Aussagen unten zustimmen.<br><br>Antwortmöglichkeiten für alle Items: *Stimme gar nicht zu / Stimme eher nicht zu / Stimme eher zu / Stimme zu*<br><br>1. Die Therapeutin/der Therapeut konnte mir gut vermitteln, wie das Online-Therapieprogramm bei "iSleep well" ablaufen wird.<br>2. Ich habe eine gute Vorstellung davon, was mich in dem Online-Therapieprogramm bei "iSleep well" zur Bewältigung meiner Schlafbeschwerden erwartet.<br>3. Die Therapeutin/der Therapeut konnte mir gut vermitteln, wie die Psychotherapie vor Ort bei "iSleep well" ablaufen wird.<br>4. Ich habe eine gute Vorstellung davon, was mich in der Psychotherapie vor Ort bei "iSleep well" zur Bewältigung meiner Schlafbeschwerden erwartet.	**Knowledge of both therapy formats**<br><br>Now we will talk about the framework conditions and contents of the two therapy formats about which you were informed in the preliminary interview and through the study materials. Please tick to what extent you agree with the statements below.<br><br>Response options for all items: *Do not agree at all / Do rather disagree / Do rather agree / Do agree*<br><br>1. The therapist was able to explain to me well how the online therapy programme at "iSleep well" will work.<br>2. I have a clear understanding of what to expect from the online therapy programme at "iSleep well" to cope with my sleep problems.<br>3. The therapist was able to effectively explain to how the on-site psychotherapy at "iSleep well" will proceed.<br>4. I have a clear understanding of what to expect from the on-site psychotherapy at "iSleep well" to cope with my sleep problems.
Original Items (German): Baseline, Post-intervention, Follow-Up	Translated Items (English): Baseline, Post-intervention, Follow-Up
**Tageslichtexposition**	**Daylight exposure**

1. Wie lange haben Sie sich in den letzten 7 Tagen durchschnittlich am Morgen (Sonnenaufgang bis 09:00 Uhr) bei Tageslicht draußen aufgehalten (ohne Dach über dem Kopf)?<br><br>Geben Sie "00:00" ein, wenn Sie es nicht wissen.<br>Dauer (als Zeit in Stunden und Minuten) _____ *(freies Textfeld)*<br><br>2. Wie lange haben Sie sich in den letzten 7 Tagen durchschnittlich am Vormittag (09:00 Uhr bis 12:00 Uhr) bei Tageslicht draußen aufgehalten (ohne Dach über dem Kopf)?<br><br>Geben Sie "00:00" ein, wenn Sie es nicht wissen.<br>Dauer (als Zeit in Stunden und Minuten) max. 3h = 180 min _____ *(freies Textfeld)*<br><br>3. Wie lange haben Sie sich in den letzten 7 Tagen durchschnittlich am Mittag (12:00 Uhr bis 15:00 Uhr) bei Tageslicht draußen aufgehalten (ohne Dach über dem Kopf)?<br><br>Geben Sie "00:00" ein, wenn Sie es nicht wissen.<br>Dauer (als Zeit in Stunden und Minuten) max. 3h = 180 min _____ *(freies Textfeld)*<br><br>4. Wie lange haben Sie sich in den letzten 7 Tagen durchschnittlich am Nachmittag (15:00 Uhr bis 18:00 Uhr) bei Tageslicht draußen aufgehalten (ohne Dach über dem Kopf)?<br><br>Geben Sie "00:00" ein, wenn Sie es nicht wissen.<br>Dauer (als Zeit in Stunden und Minuten) max. 3h = 180 min_____ *(freies Textfeld)*<br><br>5. Wie lange haben Sie in den letzten 7 Tagen durchschnittlich am Abend (18:00 Uhr bis Sonnenuntergang) bei Tageslicht draußen aufgehalten (ohne Dach über dem Kopf)?<br><br>Geben Sie "00:00" ein, wenn Sie es nicht wissen.<br>Dauer (als Zeit in Stunden und Minuten) max. 3h = 180 min_____ *(freies Textfeld)*	1. In the last 7 days, for how long on average have you been outside (without a roof over your head) in the morning (sunrise to 09.00 a.m.) in daylight?<br><br>Enter "00:00" if you do not know.<br>Duration (as time in hours and minutes) _______ *(free text field)*<br><br>2. In the last 7 days, for how long on average have you been outside (without a roof over your head) in the forenoon (09.00 a.m. to 12.00 p.m.) in daylight?<br><br>Enter "00:00" if you do not know.<br>Duration (as time in hours and minutes) max. 3h = 180 min<br>_______ *(free text field)*<br><br>3. In the last 7 days, for how long on average have you been outside (without a roof over your head) at noon (12.00 p.m. to 03.00 p.m.) in daylight?<br><br>Enter "00:00" if you do not know.<br>Duration (as time in hours and minutes) max. 3h = 180 min<br>_______ *(free text field)*<br><br>4. In the last 7 days, for how long on average have you been outside (without a roof over your head) in the afternoon (03.00 p.m. to 06.00 p.m.) during daylight hours?<br><br>Enter "00:00" if you do not know.<br>Duration (as time in hours and minutes) max. 3h = 180 min.<br>_______ *(free text field)*<br><br>5. In the last 7 days, for how long on average did you spend time outside (without a roof over your head) in the evening (6.00 p.m. to sunset) during daylight hours?<br><br>Enter "00:00" if you don't know.<br>Duration (as time in hours and minutes) max. 3h = 180 min_____ *(free text field)*
Original Items (German): Baseline, Post-intervention	Translated Items (English): Baseline, Post-intervention
**Präferenz für Therapieform (face-to-face vs online)**	**Preference for form of therapy (face-to face vs online)**

Jetzt geht es darum, welche der Behandlungsformate Sie sich für die Behandlung von Schlafbeschwerden persönlich vorstellen können.<br><br>Antwortmöglichkeiten für alle Items: *Stimme gar nicht zu / Stimme eher nicht zu / Stimme eher zu / Stimme zu*<br><br>1. Ich könnte mir gut vorstellen, das Online-Therapieprogramm zur Behandlung meiner Schlafbeschwerden zu machen.<br><br>2. Ich könnte mir gut vorstellen, die Psychotherapie vor Ort zur Behandlung meiner Schlafbeschwerden zu machen.<br><br>3. Wenn ich mich frei entscheiden könnte, würde ich mich für folgendes Therapie-Format entscheiden:<br>*o Online-Therapieprogramm mit persönlicher psychotherapeutischer Begleitung*<br>*o Psychotherapie vor Ort*<br>*o Ich habe keine Präferenz*<br><br>4. Was ist für Sie der wichtigste Grund für ein Online-Therapieprogramm?<br>_______ *(freies Textfeld)*<br><br>5. Was ist für Sie der wichtigste Grund gegen ein Online-Therapieprogramm?<br>_______ *(freies Textfeld)*	Now it's about which of the treatment formats you can personally imagine for the treatment of sleep problems.<br><br>Response options for all items: *Do not agree at all / Do rather disagree / Do rather agree / Do agree*<br><br>1. I could well imagine doing the online therapy programme to treat my sleep problems.<br><br>2. I could well imagine doing the on-site psychotherapy to treat my sleep problems.<br><br>3. If I could freely choose, I would choose the following therapy format:<br>*o Online therapy programme with face-to-face psychotherapy support*<br>*o On-site psychotherapy*<br>*o I have no preference*<br><br>4. What is the most important reason for you to choose an online therapy programme?<br>_______ *(free text field)*<br><br>5. What is the most important reason for you to not choose an online therapy programme?<br>_______ *(free text field)*
**Vorteile von Online-Therapieprogrammen**<br><br>Online-Therapieprogrammen für Schlafbeschwerden werden verschiedene Vorteile gegenüber Psychotherapie vor Ort zugeschrieben. Es folgen Aussagen über mögliche Vorteile von Online-Therapieprogrammen für Schlafbeschwerden. Bitte geben Sie nachfolgend an, wie sehr Sie persönlich diesen Aussagen zustimmen. Ich denke es ist vorteilhaft, ...<br><br>Antwortmöglichkeiten für alle Items: *Stimme nicht zu / Stimme eher nicht zu / Stimme eher zu / Stimme zu*<br><br>1. ... dass ich mich durch solch ein Online-Therapieprogramm in meiner eigenen Geschwindigkeit arbeiten kann.<br><br>2. ... dass ich selbst zeitlich flexibel entscheiden kann, wann ich solch ein Online-Therapieprogramm nutze.	**Benefits of online therapy programmes**<br><br>Online therapy programmes for sleep problems are attributed with several advantages over on-site psychotherapy. Following are statements about potential benefits of online therapy programmes for sleep problems. Please indicate below how much you personally agree with these statements. I think it is beneficial ...<br><br>Response options for all items*: Do not agree / Do rather disagree / Do rather agree / Do agree*<br><br>1. ... that I can work at my own pace through such an online therapy programme.<br><br>2. ... that I can flexibly decide for myself when to use such an online therapy programme.

3. ... dass ich das Online-Therapieprogramm örtlich flexibel nutzen kann.<br><br>4. ... dass ich das Online-Therapieprogramm an einem Ort nutzen kann, an dem ich mich wohlfühle (z. B. Sofa im Wohnzimmer, Lieblings-Parkbank).<br><br>5. ... dass ich solch ein Online-Therapieprogramm durch die Flexibilität besser mit meinen anderen Terminen in Einklang bringen kann.<br><br>6. ... dass ich selbst entscheiden kann, auf welche Inhalte und Übungen ich einen Schwerpunkt setze.<br><br>7. ... dass ich jederzeit auf alle Inhalte und selbst Erarbeitetes zurückgreifen kann und somit eine Art persönliches Therapienotizbuch habe.<br><br>8. ... dass keine Wegezeiten auf mich zukommen, weil keine An- und Abreisewege beispielsweise in eine Praxis oder Klinik entstehen.<br><br>9. ... dass ich weniger Geld für den Hin- und Rückweg zur Praxis oder Klinik ausgeben muss (z. B. Bahn-Ticket, Benzin, Parken etc.).<br><br>10. ... dass ich bei schwierigen Punkten erst einmal in Ruhe nachdenken kann ohne den Druck zu verspüren, dass jemand auf eine direkte Antwort wartet.<br><br>11. ... dass ich so oft, wie ich möchte, die schriftlichen therapeutischen Rückmeldungen nachlesen kann.<br><br>12. ... dass ich anonymer eine Therapie machen kann, weil ich weniger in einer Praxis oder Klinik sein muss, wo ich andere Menschen treffen könnte, die mitbekommen, dass ich wegen Schlafbeschwerden eine Therapie mache.<br><br>13. ... dass ich eine weitgehend digitale Therapie machen kann, die gut zu einem digitalen Lebensstil passt.<br><br>Gibt es für Sie noch weitere mögliche Vorteile eines Online-Therapieprogramms?<br><br>_______ *(freies Textfeld)*	3. ... that I can use the online therapy programme flexibly concerning location.<br><br>4. ... that I can use the online therapy programme in a place where I feel comfortable (e. g. sofa in the living room, favourite park bench).<br><br>5. ... that I can better reconcile such an online therapy programme with my other appointments because of the flexibility.<br><br>6. ... that I can decide for myself on which content and exercises I want to focus on.<br><br>7. ... that I can access all contents and what I have worked on at any time and thus have a kind of personal therapy notebook.<br><br>8. ... that I don't have to spend time traveling because I don't have to go to and from a practice or clinic, for example.<br><br>9. ... that I have to spend less money for commuting to and from the practice or clinic (e.g. train ticket, gasoline, parking fees, etc.).<br><br>10. ... that I can first think about difficult points calmly without feeling the pressure of someone waiting for a direct answer.<br><br>11. ... that I can read the written therapeutic feedback as often as I like.<br><br>12. ... that I can do therapy more anonymously, because I don't have to be in a practice or clinic as often, where I could meet other people who might notice that I am doing therapy because of sleep problems.<br><br>13. ... that I can do a largely digital therapy that fits well with a digital lifestyle.<br><br>Are there any other possible benefits of an online therapy programme for you?<br>_______ *(free text field)*
**Unerwünschte Auswirkungen von Online-Therapieprogrammen**<br><br>Online-Therapieprogramme für Schlafbeschwerden können möglicherweise auch unerwünschte Auswirkungen gegenüber Psychotherapie vor Ort haben. Es folgen Aussagen	**Adverse effects of online therapy programmes**<br><br>Online therapy programmes for sleep problems may also potentially have adverse effects compared to on-site psychotherapy. The following

über mögliche unerwünschte Auswirkungen von Online-Therapieprogrammen für Schlafbeschwerden. Bitte geben Sie nachfolgend an, für wie wahrscheinlich Sie diese Aussagen halten. Die unerwünschte Auswirkung, ...	statements outline potential adverse effects of online therapy programmes for sleep problems. Please indicate below how likely you think these statements are. The adverse effect, ...
Antwortmöglichkeiten für alle Items: *Sehr unwahrscheinlich / Eher unwahrscheinlich / Eher wahrscheinlich / Sehr wahrscheinlich*	Response options for all items*: Very unlikely / Somewhat unlikely / Somewhat likely / Very likely*
1. ... dass ich die Therapie schleifen lassen könnte, weil ich ohne feste Gesprächstermine ein Online-Therapieprogramm als weniger verbindlich empfinde.	1. ... that I might let therapy slide because I find an online therapy programme with no fixed appointment schedule less binding.
2. ... dass ich die Inhalte im Online-Therapieprogramm (z. B. Anleitungen zu Übungen oder schriftliche Rückmeldungen der Therapeutin/des Therapeuten) missverstehen könnte.	2. ... that I could misunderstand the content of the online therapy programme (e.g., instructions for exercises or written feedback from the therapist).
3. ... dass meine Nachfragen nur zeitverzögert beantwortet werden und meine Therapie dadurch ins Stocken geraten könnte.	3. ... that my queries might only be answered with time delay and that my therapy could therefore be stalled.
4. ... dass ich technische Schwierigkeiten haben könnte, die ich nicht allein lösen kann.	4. ... that I might have technical difficulties which I cannot solve on my own.
5. ... dass ich mich ohne Gesprächstermine einsam fühlen könnte.	5. ... that I could feel lonely without talking appointments.
6. ... dass meine individuellen Probleme und Schwierigkeiten weniger Raum haben könnten.	6. ... that there could be less room for my individual problems and difficulties.
7. ... dass mir das Online-Therapieprogramm in Krisen weniger ein Gefühl von Sicherheit geben könnte.	7. ... that in crises, the online therapy programme could give me less of a sense of security.
8. ... dass ich mich ohne den Kontakt vor Ort weiter zurückziehen könnte.	8. ... that I might further withdraw without the on-site contact.
9. ... dass ich weniger von dem Online-Therapieprogramm profitieren könnte, falls ich nicht so gut lesen kann.	9. ... that I might benefit less from the online therapy programme if I can't read that well.
10. ... dass ich weniger von dem Online-Therapieprogramm profitieren könnte, falls ich mich nicht so gut schriftlich ausdrücken kann.	10. ... that I might benefit less from the online therapy programme if I cannot express myself so well in writing.
11. ... dass meine Motivation für schwierige Übungen ohne direktes Gespräch geringer sein könnte.	11. ... that my motivation for difficult exercises might be lower without direct conversation.
12. ... dass ich zu Hause nicht die Ruhe finden könnte, um gewinnbringend mit dem Online-Therapieprogramm zu arbeiten.	12. ... that I might not find the peace and quiet at home to work profitably with the online therapy programme.
13. ... dass ich mich wegen Bedenken hinsichtlich der Datensicherheit und meiner Privatsphäre nicht gut auf das Online-Therapieprogramm einlassen könnte.	13. ... that I might not engage well with the online therapy programme because of concerns about data security and my privacy.

14. ... dass die Beziehung zu meiner Therapeutin/ meinem Therapeuten nicht so gut sein könnte wie bei Psychotherapie vor Ort.<br><br>Gibt es für Sie noch weitere mögliche unerwünschte Auswirkungen, die Sie ergänzen möchten?<br>_______ *(freies Textfeld)*	14. ... that the relationship with my therapist might not be as good as with on-site psychotherapy.<br><br>Are there any other possible undesirable effects for you that you would like to add?<br>_______ *(free text field)*
Original Items (German): During intervention	Translated Items (English): During intervention
**Schlaftagebuch: Morgenprotokoll**<br>(erhoben jeden Tag in der 1. bis 6. Sitzung/Lektion)<br><br>Wie erholsam war Ihr Schlaf?<br>*o Sehr*<br>*o Ziemlich*<br>*o Mittelmäßig*<br>*o Kaum*<br>*o Gar nicht*<br><br>Wie fühlen Sie sich jetzt?<br>*o Unbeschwert*<br>*o Ziemlich unbeschwert*<br>*o Eher unbeschwert*<br>*o Ziemlich bedrückt*<br>*o Bedrückt*<br><br>Wann sind Sie zu Bett gegangen? (Uhrzeit, z. B. 22:30 Uhr)<br>_______ *(freies Textfeld)*<br><br>Wie lange hat es nach dem Lichtlöschen gedauert, bis Sie eingeschlafen sind (geschätzt)? (Zeit in Stunden und Minuten, z. B. 00:05 für 5 Minuten)<br>_______ *(freies Textfeld)*<br><br>Waren Sie nachts wach?<br>___ *Ja* ___ *Nein*<br><br>Wie oft waren Sie nachts wach? (z. B. 1 für 1 Mal)<br>_______ *(freies Textfeld)*<br><br>Wie lange waren Sie insgesamt nachts wach? (Zeit in Stunden und Minuten, z. B. 00:30 für 30 Minuten)	**Sleep diary: Morning protocol**<br>(assessed every day in sessions/lessons 1 to 6)<br><br>How restful was your sleep?<br>*o Very*<br>*o Fairly*<br>*o Moderately*<br>*o Barely*<br>*o Not at all*<br><br>How do you feel now?<br>*o Carefree*<br>*o Quite carefree*<br>*o Rather carefree*<br>*o Quite depressed*<br>*o Depressed*<br><br>What time did you go to bed? (time, e.g. 10.30 p.m.)<br>_______ *(free text field)*<br><br>How long did it take until you fell asleep after turning out the light (estimated)? (Time in hours and minutes, e.g. 00:05 for 5 minutes)<br>_______ *(freies Textfeld)*<br><br>Were you awake during the night?<br>___ *Yes* ___ *No*<br><br>How many times were you awake during the night? (e.g. 1 for 1 time)<br>_______ *(free text field)*<br><br>How long were you awake at night in total? (time in hours and minutes, e.g. 00:30 for 30 minutes)

_______ *(freies Textfeld)*	_______ *(free text field)*
Wann sind Sie endgültig aufgewacht? (Uhrzeit, z. B. 07:00 Uhr)<br>_______ *(freies Textfeld)*	What time did you finally wake up? (time, e.g. 07.00 a.m.)<br>_______ *(free text field)*
Wann sind Sie endgültig aufgestanden? (Uhrzeit, z. B. 07:15 Uhr)<br>_______ *(freies Textfeld)*	What time did you finally get up? (time, e.g. 07.15 a.m.)<br>_______ *(free text field)*
Wie lange haben Sie insgesamt geschlafen? (Zeit in Stunden und Minuten, z. B. 08:15 für 8 Stunden und 15 Minuten)<br>_______ *(freies Textfeld)*	How long did you sleep in total? (time in hours and minutes, e.g. 08:15 for 8 hours and 15 minutes)<br>_______ *(free text field)*
Haben Sie gestern Abend Schlafmittel (auch freiverkäufliche) eingenommen?<br>___ *Ja* ___ *Nein*	Did you take any sleeping pills (including over-the-counter) last night?<br>___ *Yes* ___ *No*
Welches Schlafmittel haben Sie eingenommen? (z. B. Ximovan) _______ *(freies Textfeld)*	Which sleeping pill did you take? (e.g. Ximovan) _______ *(free text field)*
Falls Sie mehrere Schlafmittel eingenommen haben, nennen Sie hier bitte zunächst eines.<br>_______ *(freies Textfeld)*	If you have taken more than one kind of sleeping pills, please name one first. _______ *(free text field)*
In welcher Dosis haben Sie das Schlafmittel eingenommen? (z. B. 1 Tablette oder 10 mg)<br>_______ *(freies Textfeld)*	In which dose did you take the sleeping pill? (e.g. 1 tablet or 10 mg)<br>_______ *(free text field)*
Um wie viel Uhr haben Sie das Schlafmittel eingenommen? (Uhrzeit, z. B. 21:00 Uhr)<br>_______ *(freies Textfeld)*	At what time did you take the sleeping pill? (time, e.g. 09.00 p.m.)<br>_______ *(free text field)*
Haben Sie gestern Abend ein weiteres Schlafmittel eingenommen?<br>___ *Ja* ___ *Nein*	Did you take another sleeping pill last night?<br>___ *Yes* ___ *No*
Falls Ja: Welches Schlafmittel haben Sie eingenommen? _______ *(freies Textfeld)*	If Yes: Which sleeping pill did you take? _______ *(free text field)*
In welcher Dosis haben Sie das Schlafmittel eingenommen? _______ *(freies Textfeld)*	In what dosage did you take the sleeping pill? _______ *(free text field)*
Um wie viel Uhr haben Sie das Schlafmittel eingenommen?<br>Haben Sie gestern Abend ein weiteres Schlafmittel eingenommen?<br>___ *Ja* ___ *Nein*	At what time did you take the sleeping pill?<br>Did you take another sleeping pill last night?<br>___ *Yes* ___ *No*
Falls Ja: Welches Schlafmittel haben Sie eingenommen?<br>In welcher Dosis haben Sie das Schlafmittel eingenommen?<br>(z. B. 1 Tablette oder 10 mg) _______ *(freies Textfeld)*	If Yes: Which sleeping pill did you take?<br>In what dosage did you take the sleeping pill?<br>(e.g. 1 tablet or 10 mg) _______ *(free text field)*
Um wie viel Uhr haben Sie das Schlafmittel eingenommen?<br>(Uhrzeit, z. B. 21:00 Uhr) _______ *(freies Textfeld)*	At what time did you take the sleeping pill?<br>(time, e.g. 09.00 p.m.) _______ *(free text field)*

**Schlaftagebuch: Abendprotokoll**<br>(erhoben jeden Tag inder 1. bis 6. Sitzung/Lektion)	**Sleep diary: Evening protocol**<br>(assessed every day in sessions/lessons 1 to 6)
Wie fühlen Sie sich jetzt?<br>*o Entspannt*<br>*o Ziemlich entspannt*<br>*o Eher entspannt*<br>*o Eher angespannt*<br>*o Ziemlich angespannt*<br>*o Angespannt*	How do you feel now?<br>*o Relaxed*<br>*o Quite relaxed*<br>*o Rather relaxed*<br>*o Rather tense*<br>*o Fairly tense*<br>*o Tense*
Wie war heute Ihre durchschnittliche Leistungsfähigkeit?<br>*o Gut*<br>*o Ziemlich gut*<br>*o Eher gut*<br>*o Eher schlecht*<br>*o Ziemlich schlecht*<br>*o Schlecht*	What was your average performance level today?<br>*o Very good*<br>*o Rather good*<br>*o Good*<br>*o Bad*<br>*o Rather bad*<br>*o Very bad*
Haben Sie sich heute erschöpft gefühlt?<br>*o Nein*<br>*o Ein wenig*<br>*o Ziemlich*<br>*o Sehr*	Did you feel exhausted today?<br>*o No*<br>*o A little bit*<br>*o Quite*<br>*o Very much*
Haben Sie heute tagsüber geschlafen?<br>___ *Ja* ___ *Nein*	Did you sleep during the day today?<br>___ *Yes* ___ *No*
Wie lange haben Sie heute insgesamt tagsüber geschlafen? (Zeit in Stunden und Minuten, z. B. 00:20 für 20 Minuten) _______ *(freies Textfeld)*	How long did you sleep during the day in total? (time in hours and minutes, e.g. 00:20 for 20 minutes). _______ *(free text field)*
Wann sind Sie von Ihrem (letzten) Mittagsschlaf aufgewacht? (Uhrzeit, z. B. 15:30 Uhr)<br>_______ *(freies Textfeld)*	At what time did you wake up from your (last) afternoon nap? (time, e.g. 03.30 p.m.) _______ *(free text field)*
Haben Sie heute geraucht? (z. B. Zigarette, E-Zigarette, Pfeife)<br>___ *Ja* ___ *Nein*	Did you smoke today? (e.g. cigarette, e-cigarette, pipe)<br>___ *Yes* ___ *No*
Wie viel haben Sie heute geraucht? (z. B. 1 Zigarette) _______ *(freies Textfeld)*	How much did you smoke today? (e.g. 1 cigarette)<br>_______ *(free text field)*
Wann haben Sie heute das letzte Mal geraucht? (Uhrzeit, z. B. 21:30 Uhr)<br>_______ *(freies Textfeld)*	When was the last time you smoked today? (time, e.g. 9.30 p.m.)<br>_______ *(free text field)*

Haben Sie heute 4 Stunden vor dem Zu-Bett-Gehen Alkohol zu sich genommen?<br>___ *Ja* ___ *Nein*<br><br>Was für Alkohol haben Sie in diesem Zeitraum zu sich genommen und wie viel? (z. B. 0,2 l Bier) _______ *(freies Textfeld)*<br><br>Haben Sie (darüber hinaus) heute 8 Stunden vor dem Zu-Bett-Gehen Alkohol zu sich genommen?<br>___ *Ja* ___ *Nein*<br><br>Was für Alkohol haben Sie in diesem Zeitraum zu sich genommen und wie viel? (z. B. 0,1 l Sekt) _______ *(freies Textfeld)*<br><br>Haben Sie heute 4 Stunden vor dem Zu-Bett-Gehen koffeinhaltige Getränke zu sich genommen?<br>___ *Ja* ___ *Nein*<br><br>Was für koffeinhaltige Getränke haben Sie in diesem Zeitraum zu sich genommen und wie viel? (z. B. 0,2 l Cola) _______ *(freies Textfeld)*<br><br>Haben Sie (darüber hinaus) heute 8 Stunden vor dem Zu-Bett-Gehen koffeinhaltige Getränke zu sich genommen?<br>___ *Ja* ___ *Nein*<br><br>Was für koffeinhaltige Getränke haben Sie in diesem Zeitraum zu sich genommen und wie viel? (z. B. 2 Tassen Kaffee) _______ *(freies Textfeld)*	Did you drink alcohol today 4 hours before going to bed?<br>___ *Yes* ___ *No*<br><br>What kind of alcohol did you drink during this period and how much? (e.g. 0.2 l beer) _______ *(free text field)*<br><br>Did you drink alcohol (in addition) today 8 hours before going to bed?<br>___ *Yes* ___ *No*<br><br>What kind of alcohol did you drink during this period and how much? (e.g. 0.1 l sparkling wine) _______ *(free text field)*<br><br>Did you drink caffeinated beverages today 4 hours before going to bed?<br>___ *Yes* ___ *No*<br><br>What kind of caffeinated drinks did you have during this period and how much? (e.g. 0.2 l cola) _______ *(free text field)*<br><br>Did you drink caffeinated beverages (in addition) today 8 hours before going to bed?<br>___ *Yes* ___ *No*<br><br>What kind of caffeinated drinks did you drink during this period and how many? (e.g. 2 cups of coffee) _______ *(free text field)*
**Unerwünschte Auswirkungen und Nebenwirkungen** (erhoben in der 2. bis 6. Sitzung/Lektion)<br><br>Sind in den vergangenen Tagen bei Ihnen unerwünschte Auswirkungen bzw. Nebenwirkungen aufgetreten, die Sie im Zusammenhang mit dem Online-Therapieprogramm sehen?<br>_______ *(freies Textfeld)*	**Adverse effects and side effects** (assessed in sessions/lessons 2 to 6)<br><br>In the last days, have there been adverse effects or side effects that occurred in connection with the online therapy programme?<br>_______ *(free text field)*
**Veränderung von Behandlungen (Medikation und Psychotherapie)**<br>(erhoben in der 4. und 6. Sitzung/Lektion)<br><br>Sie werden sich bestimmt daran erinnern, dass Ihre Psychotherapeutin/ Ihr Psychotherapeut Sie im Erstgespräch nach Ihrer Medikamenteneinnahme gefragt hat. Also ob Sie regelmäßig Medikamente gegen Ihre Schlafbeschwerden (auch frei verkäufliche) einnehmen.	**Changes in treatments (medication and psychotherapy)**<br>(assessed in sessions/lessons 4 and 6)<br><br>You can surely remember that, in the first meeting, your psychotherapist asked you about your medication intake, whether you are regularly taking medication for your sleep problems (including over-the-counter medication).

Zu Auswertungszwecken ist es wichtig, dass Sie nachfolgend angeben, ob sich daran in den letzten Wochen des Online-Programms in irgendeiner Form etwas geändert hat.<br><br>Bitte kreuzen Sie dafür Zutreffendes an:<br>*o Es haben sich seit dem Erstgespräch Änderungen bzgl. der Schlafmedikation ergeben (z. B. Dosis verändert, (neues) Medikament eingenommen).*<br>*o Es haben sich seit dem Erstgespräch KEINE Änderungen bzgl. der Schlafmedikation ergeben(z. B. weiterhin gleiche Dosis oder weiterhin keine Einnahme von Schlafmedikamenten).*<br><br>Und noch eine zweite Frage: Haben Sie in den letzten Wochen des Online-Programms eine weitere Psychotherapie begonnen?<br>*o Ja, ich habe eine weitere Psychotherapie begonnen.*<br>*o Nein, ich habe KEINE weitere Psychotherapie begonnen.*	For evaluation purposes, it is important that you report any changes in your medication in the last weeks of the online programme.<br><br>Please tick the option that applies to your situation:<br>*o There have been changes in my sleep medication since the first meeting (e.g. change of dose, intake of new sleep medication)*<br>*o There have been NO changes in my sleep medication since the first meeting (e.g. change of dose, intake of new sleep medication*)<br><br>A second question: Have you started another psychotherapy in the last weeks of the online programme?<br>*o Yes, I have started another psychotherapy.*<br>*o No, I have NOT started another psychotherapy.*
Original Items (German): Post-intervention	Translated Items (English): Post-intervention
**Adhärenz (Selbstbeurteilung nach Interventionsende)**<br><br>In der Therapie haben Sie in maximal 6 Onlinetherapie-Lektionen oder maximal 6 Therapiesitzungen verschiedene Möglichkeiten kennengelernt, was Sie für einen besseren Schlaf tun können.<br><br>Wie viele Lektionen haben Sie bearbeitet (Online-Therapieprogramm) bzw. an wie vielen Therapie-Sitzungen (vor Ort) haben Sie teilgenommen? _______ (*0-6, Anzahl eintragen*)<br><br>Manche Patientinnen/Patienten können besser von den einen Übungen profitieren, andere bevorzugen es, andere Empfehlungen aus der Therapie in ihrem Alltag anzuwenden. Wir möchten gerne von Ihnen erfahren, welche der Übungen und Empfehlungen Sie genutzt haben.<br><br>Es geht im Folgenden darum, wie oft Sie verschiedene Übungen aus dem Therapieprogramm in den letzten 2 Wochen umgesetzt haben.<br><br>Wie oft sind Sie in den letzten 2 Wochen... (Antwortmöglichkeiten für alle Items: *nie oder selten / gelegentlich / an der Hälfte der Tage / an den meisten Tagen / jeden Tag*).<br><br>1. ... so lange wachgeblieben, wie Sie nach Ihrem aktuellen, selbst errechneten (oder dem letzten in der Therapie festgelegten) Bettzeitfenster wachbleiben sollten (maximal 15 Minuten früher ins Bett gegangen)?	**Adherence (self-assessment after treatment)**<br><br>In therapy, you have learned about different options you can use for better sleep in a maximum of 6 online therapy lessons or a maximum of 6 therapy sessions.<br><br>How many lessons did you work on (online therapy programme) or how many therapy sessions (on-site) did you attend? _______ (*0-6, fill in the number*)<br><br>Some patients may benefit more from certain exercises while others prefer to apply other recommendations from therapy in their daily lives. We would like to hear from you which of the exercises and recommendations you have used.<br><br>The following is about how often you have implemented different exercises from the therapy programme in the last 2 weeks.<br><br>In the last 2 weeks, how often did you...? (Response options for all items: *never or rarely / occasionally / half the days / most days / every day*).<br><br>1. ... stay awake as long as you were supposed to according to your current, self-calculated (or in therapy established) sleep window (went to bed no more than 15 minutes earlier)?

2. ... zu der Uhrzeit aufgestanden, zu der Sie nach Ihrem aktuellen, selbst errechneten (oder letzten in der Therapie festgelegten) Bettzeitfenster aufstehen sollten (maximal 15 Minuten später aufgestanden)?<br><br>3. ... erst ins Bett gegangen, als Sie schon sehr müde waren?<br><br>4. ... nachts oder morgens aufgestanden und haben das Bett verlassen, wenn Sie nach gefühlten 15 Minuten nicht wieder einschlafen konnten?<br><br>Wie oft haben Sie in den letzten 2 Wochen... (Antwortmöglichkeiten für alle Items: *nie oder selten / gelegentlich / an der Hälfte der Tage / an den meisten Tagen / jeden Tag*)<br><br>5. ... tagsüber geschlafen?<br><br>6. ... Ihr Bett nur zum Schlafen (und eventuell Sexualität) genutzt?<br><br>7. ... Entspannungsübungen gemacht (z. B. Progressive Muskelentspannung (PMR), Bodyscan, Ruhebild)?<br><br>8. ... Übungen zu schlafhinderlichen Gedanken, Sorgen und Grübeln gemacht, um besser schlafen zu können (z. B. Gelassenheitsgedanke, Gedankenstuhl, Gedankentagebuch und Imaginationsübungen zum Gedanken ziehen lassen).<br><br>9. ... Schlafhygiene-Regeln umgesetzt (z. B. nachmittags keine koffeinhaltigen Getränke, abends keine alkoholischen Getränke, angenehme Schlafatmosphäre, Entspannung vor Schlaf)?	2. ... got up at the time you were supposed to get up according to your current, self-calculated (or in therapy established) bedtime window (got up no more than 15 minutes later)?<br><br>3. ... not go to bed until you were very tired?<br><br>4. ... got up at night or in the morning and got out of bed when you could not fall back asleep after what felt like 15 minutes?<br><br>In the last 2 weeks, how often did you... (Response options for all items: *never or rarely / occasionally / half the days / most days / every day*).<br><br>5. ... sleep during the day?<br><br>6. ... use your bed only for sleeping (and possibly sex)?<br><br>7. ... do relaxation exercises (e.g., progressive muscle relaxation (PMR), body scan, imagination exercise: mental image of tranquility)?<br><br>8. ... do exercises on sleep-preventing thoughts, worries, and ruminations to help sleep better (e.g., “thought of serenity”, worry chair, thought diary, and imagination exercises to let thoughts pass)?<br><br>9. ... implement sleep hygiene rules (e.g. no caffeinated beverages in the afternoon, no alcoholic beverages in the evening, pleasant sleeping atmosphere, relaxation before sleep)?

Appendix 2: Self-developed questionnaire items for therapists

Original Items (German): Assessed in sessions/lessons 1 to 6	Translated Items (English): Assessed in sessions/lessons 1 to 6
**Adhärenz während laufender Therapie**<br><br>In der Therapie haben die Patient:innen verschiedene Methoden und Techniken kennengelernt, mit denen sie ihren Schlaf verbessern können. Um den Zusammenhang zwischen Therapieadhärenz und Wirksamkeit der KVT-I besser zu verstehen, möchten wir Sie bitten, wöchentlich eine Frage zu Ihrem persönlichen Eindruck zu beantworten, inwiefern die Patientin / der Patient Übungen des Therapieprogramms umsetzt.<br><br>Ich habe den Eindruck, dass die Patientin / der Patient eine ausreichende Anzahl von zur Symptomatik passenden Übungen der KVT-I zwischen den Therapiesitzungen bzw. Lektionen umsetzt (z. B. Schlafrestriktion, Stimuluskontrolle, Schlafhygiene, Entspannungsübungen, kognitive Techniken).<br>_______ *(0 = hat gar keine Übungen umgesetzt – 10 = hat eine ausreichende Anzahl von Übungen umgesetzt)*<br><br>Zudem möchten wir die Therapieadhärenz speziell hinsichtlich der Bettzeitrestriktion berechnen. Wir wollen dafür das vereinbarte Bettzeitfenster mit den Zeiten vergleichen, die die Patient:innen im Schlaftagebuch eintragen.<br><br>Bitte tragen Sie hier das mit dem Patienten / der Patientin festgelegte Bettzeitfenster ein und ab welchem Datum es eingehalten werden soll (vor Ort = der Tag, an dem die Sitzung stattgefunden hat; Online = der Tag, an dem Sie die Rückmeldung dazu geschrieben haben).<br>Datum, von bis _______ *(freies Textfeld)*<br><br>Wie viel Zeit haben Sie diese Woche für die Therapie dieser Patientin / dieses Patienten insgesamt aufgewandt? Dazu zählen insbesondere die Vor- und Nachbereitung der Therapiesitzung und Dauer der Therapiesitzung (vor Ort) bzw. das Lesen von Eintragungen und schriftliches Feedback dazu (Online) sowie das Beantworten von Nachrichten der Patientin/des Patienten (beide Therapieformate).	**Adherence during treatment**<br><br>In therapy, patients have learned different methods and techniques to improve their sleep. To better understand the relationship between therapy adherence and effectiveness of CBT-I, we would like to ask you to answer one question each week about your personal impression of the extent to which the patient implements exercises of the therapy programme.<br><br>I have the impression that the patient implements a sufficient number of exercises of CBT-I that match his/her symptoms between therapy sessions or lessons (e. g. sleep restriction, stimulus control, sleep hygiene, relaxation exercises, cognitive techniques).<br>_______ *(0 = did not implement any exercises at all – 10 = implemented a sufficient number of exercises)*<br><br>In addition, we would like to calculate therapy adherence specifically with respect to bedtime restriction. For this, we want to compare the agreed bedtime window with the times that the patients enter in the sleep diary.<br><br>Please enter here the bedtime window agreed upon with the patient and from which date it should be observed (on-site = the day the session took place; online = the day you wrote the feedback on it).<br>Date, from to _______ *(free text field)*<br><br>How much time in total did you spend on this patient's therapy this week? This includes in particular the preparation and follow-up of the therapy session and duration of the therapy session (on-site) or reading entries and written feedback on them (online) as well as answering messages from the patient (both therapy formats).
